# Sirtuin 5 aggravates microglia-induced neuroinflammation following ischaemic stroke by modulating the desuccinylation of Annexin-A1

**DOI:** 10.1186/s12974-022-02665-x

**Published:** 2022-12-14

**Authors:** Qian Xia, Shuai Gao, Tangrui Han, Meng Mao, Gaofeng Zhan, Yonghong Wang, Xing Li

**Affiliations:** 1 Department of Anesthesiology, Tongji Hospital, Tongji Medical College, Huazhong University of Science and Technology, Wuhan, 430030 China; 2 Department of Neurosurgery, Shanxi Bethune Hospital, Shanxi Academy of Medical Sciences, Tongji Shanxi Hospital, Third Hospital of Shanxi Medical University, Taiyuan, 030032 China; 3 Department of Anesthesiology and Perioperative Medicine, Zhengzhou Central Hospital Affiliated to Zhengzhou University, Zhengzhou, 450007 China

**Keywords:** Sirtuin 5, Annexin A1, Succinylation, Microglia, Neuroinflammation, Cerebral ischaemia and reperfusion injury

## Abstract

**Background:**

Microglia-induced excessive neuroinflammation plays a crucial role in the pathophysiology of multiple neurological diseases, such as ischaemic stroke. Controlling inflammatory responses is considered a promising therapeutic approach. Sirtuin 5 (SIRT5) mediates lysine desuccinylation, which is involved in various critical biological processes, but its role in ischaemic stroke remains poorly understood. This research systematically explored the function and potential mechanism of SIRT5 in microglia-induced neuroinflammation in ischaemic stroke.

**Methods:**

Mice subjected to middle cerebral artery occlusion were established as the animal model, and primary cultured microglia treated with oxygen–glucose deprivation and reperfusion were established as the cell model of ischaemic stroke. SIRT5 short hairpin RNA, adenovirus and adeno-associated virus techniques were employed to modulate SIRT5 expression in microglia both in vitro and in vivo. Coimmunoprecipitation, western blot and quantitative real-time PCR assays were performed to reveal the molecular mechanism.

**Results:**

In the current study, we showed that SIRT5 expression in microglia was increased in the early phase of ischaemic stroke. SIRT5 interacts with and desuccinylates Annexin A1 (ANXA1) at K166, which in turn decreases its SUMOylation level. Notably, the desuccinylation of ANXA1 blocks its membrane recruitment and extracellular secretion, resulting in the hyperactivation of microglia and excessive expression of proinflammatory cytokines and chemokines, ultimately leading to neuronal cell damage after ischaemic stroke. Further investigation showed that microglia-specific forced overexpression of SIRT5 worsened ischaemic brain injury, whereas downregulation of SIRT5 exhibited neuroprotective and cognitive-preserving effects against ischaemic brain injury, as proven by the decreased infarct area, reduced neurological deficit scores, and improved cognitive function.

**Conclusions:**

Collectively, these data identify SIRT5 as a novel regulator of microglia-induced neuroinflammation and neuronal damage after cerebral ischaemia. Interventions targeting SIRT5 expression may represent a potential therapeutic target for ischaemic stroke.

**Supplementary Information:**

The online version contains supplementary material available at 10.1186/s12974-022-02665-x.

## Background

Ischaemic stroke is one of the leading causes of disability and death throughout the world [1, 2]. Brain injury after cerebral ischaemia involves complex pathological mechanisms [3]. In the past decade, much attention has been focused on exploring effective neuroprotective strategies targeting only neurons, which cannot produce better outcomes postinjury [4]. Accumulating studies have revealed that microglial activation and neuroinflammation play crucial roles in the pathophysiology of cerebral ischaemia [5, 6]. Current evidence suggests that hyperactivated microglia induced by cerebral ischaemia and reperfusion (I/R) injury can secrete a variety of proinflammatory cytokines and chemokines, including interleukin (IL)-1β, tumour necrosis factor (TNF)-α, interleukin (IL)-6, C-X-C motif chemokine ligand 1 (CXCL1), and C–C motif chemokine ligand 2 (CCL2), which are essential for the fate of bystander neurons in the penumbra and result in delayed deterioration of ischaemic tissue [7]. Accordingly, research on relevant targets to restrain neuroinflammation and attenuate the release of inflammatory factors by microglia could potentially suppress neuronal damage and contribute to the treatment of ischaemic stroke.

Annexin A1 (ANXA1) is a well-recognized 37-kDa member of the annexin superfamily involved in a variety of important biological processes [8–12]. In the central nervous system (CNS), ANXA1 is abundant in microglial cells [13]. Compelling evidence shows that ANXA1 plays a crucial role in microglia-induced inflammatory processes [14], and the role of ANXA1 in the inflammatory response depends on its subcellular protein localization. A previous study revealed that ANXA1 can be transported to the plasma membrane, and once on the cell surface, ANXA1 can act in an autocrine (or paracrine) fashion to inhibit microglial hyperactivation by interacting with receptors of the FPR family, specifically FPR2 [13]. However, our previous research also reported that ANXA1 could be translocated into the nucleus and induce excessive production of proinflammatory factors after oxygen–glucose deprivation and reperfusion (OGD/R) injury [15]. Numerous studies have claimed that posttranslational modifications (PTMs), such as phosphorylation and SUMOylation, strictly regulate the biological function of ANXA1 [16, 17]. For example, our recent study demonstrated that SENP6-mediated deSUMOylation of ANXA1 could induce its nuclear translocation and trigger neuronal apoptosis after ischaemic stroke [18]. Beyond SUMOylation modifications, a global lysine succinylation proteome analysis revealed that ANXA1 can also be modified by succinyl-CoA in a process termed succinylation [19]. However, the role of ANXA1 succinylation and the key enzymes which regulate the succinylation status of ANXA1 during ischaemic stroke remain poorly understood.

Large-scale proteomic surveys have demonstrated that cellular proteins are subject to multiple reversible PTMs [20]. Examples of such modifications include phosphorylation, acetylation, succinylation, SUMOylation, and glycosylation. Among them, succinylation is a relatively recently discovered novel PTM that may be implicated in numerous biological functions, including the control of ageing, cell proliferation, differentiation, apoptosis, inflammatory reaction and activation of metabolic pathways [19, 21, 22]. Sirtuins are an evolutionarily conserved family of NAD-dependent lysine deacylases and include seven mammalian sirtuins (SIRT1–7) [23]. Unlike other sirtuins, SIRT5 and SIRT7 possess very weak deacetylase activities but have unique enzyme activity, such as lysine succinylation [24]. A variety of (pathological) physiological functions of SIRT5 are currently emerging [25, 26]. A previous study reported that SIRT5 increases blood–brain barrier (BBB) permeability by degrading occludin, thereby mediating I/R-induced brain damage [27]. SIRT5 was also reported to have protective effects to decrease the infarct size following myocardial I/R injury [28]. Although the effects of SIRT5 on the CNS have been introduced, whether SIRT5 also functions in the pathophysiology of ischaemic stroke remains poorly characterized.

Accordingly, the current research aims to explore the pathological role of SIRT5 in microglia-induced neuroinflammation after ischaemic stroke. To this end, we assessed the effect of genetic overexpression and knockdown of SIRT5 on I/R-induced brain damage with a specific focus on neuroinflammation and neuronal damage using in vivo and in vitro models of ischaemic stroke. We provide experimental evidence that the interaction between SIRT5 and ANXA1 increases and that SIRT5 mediates the desuccinylation of ANXA1 after ischaemic stroke. Importantly, improving SIRT5 significantly promotes ANXA1 nuclear translocation and suppresses ANXA1 membrane recruitment, followed by microglial hyperactivation, ultimately leading to excessive neuroinflammation and neuronal damage after ischaemic stroke. In contrast, suppression of SIRT5-mediated desuccinylation of ANXA1 effectively inhibits neuroinflammation and improves neurological function after ischaemic stroke. Collectively, our data identified that SIRT5 inhibition might represent a potential therapeutic strategy for ischaemic stroke.

## Materials and methods

### Animals

C57BL/6J male mice (weighing 22–25 g, aged 11–12 weeks) were obtained from Beijing Vital River Laboratory Animal Corp. Ltd. Cx3cr1-Cre mice were purchased from Jackson Laboratory (JAX stock No: 025524). All animals were maintained under certain disease-free conditions. Mice were maintained at a steady temperature of 22 °C and provided with a normal diet and free access to water. The research was approved by the IACUC of Tongji Hospital, Tongji Medical College, Huazhong University of Science and Technology. The study was conducted according to the IMPROVE guidelines [29]. The protocols and details of this report were in accordance with the ARRIVE guidelines [30]. The number of animals for each group was predetermined according to numbers reported in published studies or our prior experiment, and accurate animal numbers are given in figure legends. All animals were randomly assigned for the study and procedure. The operator was blinded to the data analysis and experimentation.

### Transient focal cerebral ischaemia

Left middle cerebral artery embolization was used to establish an ischaemic stroke animal model as described previously [31]. The entire procedure was performed on a thermostatic mat at 37 °C. Briefly, male mice were anaesthetized with an intraperitoneal injection of 4% chloral hydrate (350 mg/kg). The left external carotid artery (ECA), internal carotid artery (ICA) and common carotid artery (CCA) were exposed with a midline cervical incision. Then, the distal carotid artery was ligated, and two surgical nylon monofilaments were used to ligate the ECA: one was ligated to the far end of the ECA, and the other was ligated to the junction of the ECA and the ICA. Finally, between the ECA ligatures, we made an incision, and a 2-cm nylon filament (diameter: 0.25 ± 0.03 mm) was interposed into the middle cerebral artery ring from the incision of the ECA and through the ICA. The LCBF was measured by laser Doppler flowmetry (PeriFlux System 5010; Perimed, Jarfalla, Sweden). The nylon filament was removed after an hour of ischaemia for reperfusion. The mice were resuscitated on thermal pads for 2 h and then returned to a warm cage with food and water. The sham mice were treated in the same way but without embolus inserts.

### Cell culture and OGD/R treatment

Primary cultured neurons were isolated from mouse embryos (16–18 days) with the procedure as we previously described [32]. Primary microglia were obtained from neonatal mouse brains (1–2 days). Briefly, the cerebral cortices were gently separated under an anatomic microscope, and the cell suspension was filtered with a 70-µm pore filter. Then, the cells were resuspended in high-glucose Dulbecco’s modified Eagle’s medium (DMEM, Thermo Fisher Scientific, Waltham, MA, USA) and 10% foetal bovine serum (FBS, Gibco, Gaithersburg, MD, USA). For the primary microglia, after dissection for 10–14 days, the cells were collected by oscillating on a rotating oscillator at 37 °C at a speed of 400 rpm for 6 h. The purity of adherent cells was verified by immunocytochemical staining, which indicated more than 95% of the cells in cultures were positive for the microglia-specific marker Iba-1 (Wako, 1:500) (Additional file 4: Fig. S4A).

HEK293T cells were obtained from the American Type Culture Collection. Cells were grown in DMEM supplemented with 10% FBS and 1% penicillin–streptomycin (Beyotime, Shanghai, China) at 37 °C in a humidified 5% CO_2_-containing atmosphere. Confluent cell layers were split three times per week. Transfections of the plasmids were performed by using Lipofectamine 3000 (Invitrogen, NY, USA) when the cells were 80 to 90% confluent, following the manufacturer’s instructions.

The OGD/R procedure was conducted as previously reported [33]. Briefly, cells were cultured in DMEM with glucose containing 10% FBS and incubated at 37 °C with 5% CO_2_. After the corresponding experimental treatment, the cells were subjected to OGD treatment. Briefly, the supernatant of cultured cells was replaced with DMEM without glucose, and the cells were placed at 37 °C in an atmosphere of 1% O_2_, 94% N_2_, and 5% CO_2_. After 60 min of incubation, the cultivation medium was replaced with DMEM with glucose, and the cells were returned to the normoxic incubator (5% CO_2_ and 95% O_2_).

### Co-culture of neuron and microglia

A co-culture transwell (Corning, Shanghai, China) system were applied to separate the primary microglial cells and neuronal cells. Cells were cultured in two loculi segregated by a 0.4-μm semi-permeable membrane to allow cytokines to diffuse between the two chambers. First, the neurons were cultured in the lower compartments of the transwell, while the microglia cells were cultured in the upper chamber using the same procedure described above. After adenovirus transfected into microglia cells for 48 h infection, the supernatants were replaced with free fresh medium and the transwell insert was moved to the neurons. The microglia and neurons were co-cultured for 24 h. Then, cells were subjected to OGD/R treatment.

### Viral vector transduction

Adenovirus vectors encoding the SIRT5 gene sequence or shRNA sequences against SIRT5 were obtained from Vigene Biosciences, Jinan, China. Primary cultured microglial cells were infected with the respective adenoviruses at an optimal multiplicity of infection of 100. For in vivo viral infection studies, Cx3cr-Cre male mice (8 weeks) were transduced with AAV2/6 viruses (2–3 × 10^12^ vg/ml) encoding CMV-DIO-Vector, CMV-DIO-His-SIRT5, U6-DIO-shNC-EGFP, U6-DIO-shSIRT5-EGFP by intraperitoneal injection, which induced SIRT5 overexpression or knockdown in microglia specifically. The target sequence for mouse SIRT5 (GenBank NM_178848.3) shRNA no. 1 was 5′-ATCACGTAACAGATTGTCTGC-3′, no. 2 was 5′-ATCGGACTCCTATAGTTCTCG-3′, and a Scr shRNA served as a scramble control group. Stereotaxic surgery was conducted to inject AAV as we previously reported [14]. Briefly, the mice were anaesthetized with an intraperitoneal injection of 4% chloral hydrate (350 mg/kg) and fixed to a stereoscope (RWD Life Science, Shenzhen, China). An aperture was made with a skull drill, and 500 nL of virus (50 nL/min) solution was injected into the left hemisphere hippocampal CA1 region, cerebral cortex and striatum with a stepper motor-driven microinjector (Hamilton, Reno, USA). The coordinates were as follows: hippocampus CA1 region (AP: − 2.00 mm, ML: − 1.55 mm, DV: − 1.55 mm), cerebral cortex region (AP: 0.00 mm, ML: − 2.05 mm, DV: − 1.50 mm), and striatum region (AP: 0.14 mm, ML: − 2.28 mm, DV: − 3.50 mm). Abbreviations for virus components are as follows: DIO, double-flexed inverted open reading frame; ITR, inverted terminal repeat; U6, RNA polymerase III-dependent promoter.

### Immunofluorescence

Briefly, the cell slides were fixed with 4% paraformaldehyde for 30 min at room temperature, washed three times in PBS, and incubated with 0.1% Triton X-100 for 15 min. The slides were blocked with 5% bovine serum albumin (BSA) (Sigma-Aldrich, Shanghai, China) for 1 h at room temperature and stained with anti-SIRT5 antibody (1:50; Proteintech, Wuhan, China) in immunofluorescence buffer (10% BSA in PBS) at 4 °C overnight. Subsequently, the probed slides were incubated with FITC-conjugated secondary antibody for 2 h at room temperature. Then, the slides were incubated with 4′,6-diamidino-2-phenylindole (DAPI, Sigma-Aldrich) for 8 min at room temperature. Coverslips were mounted on the culture slides. Finally, all images were examined with a luminescence microscope (BX53, Olympus, Tokyo, Japan). In each well, 50 to 100 cells were measured using ImageJ software (NIH, Baltimore, MD, USA). The analysis was performed while investigators were blinded to the experiment.

### Western blot analysis

Total proteins were harvested from cells with ice-cold RIPA buffer (Beyotime). Lysates were centrifuged at 12,000×*g* at 4 °C for 15 min, and then the protein samples were heated at 95 °C for 5 min. SDS-PAGE gels were utilized to separate the proteins, and then the proteins were transferred onto a polyvinylidene fluoride membrane (PVDF; Roche, Basel, Switzerland). The membranes were then blocked with 5% fat-free milk for 1 h at room temperature and incubated with the primary antibodies of interest overnight at 4 °C. The secondary antibodies were anti-rabbit IgG and anti-mouse IgG conjugated to HRP. Finally, the protein bands were developed by chemiluminescence detection (ECL; Thermo Fisher Scientific) and then analysed by band densitometry with ImageJ software. The relative levels of these proteins were normalized to β-actin. All antibodies are listed in Additional file 7: Table S1.

### RNA extraction and quantitative real-time PCR (qPCR)

Total RNA was isolated from samples using TRIzol reagent (Invitrogen, Carlsbad, CA, USA) following the protocol provided by the manufacturer. Afterwards, cDNA was synthesized from 1 μg of RNA using the ReverTra Ace-α-TM First Strand cDNA Synthesis Kit (Toyobo, Osaka, Japan). The cDNA was then analysed using real‐time qPCR with SYBR Green PCR Master mix (Toyobo) on a StepOnePlus™ Real‐Time PCR System (Applied Biosystems, Foster City, USA) according to the manufacturer’s instructions. The 2^−ΔΔCt^ method was utilized to analyse gene expression, and relative mRNA expression values were normalized to β*-actin*. The primers for quantitative real-time PCR assays are listed in Additional file 7: Table S2.

### Morris water maze (MWM) test

The spatial learning and memory of the mice were determined by the MWM test as described previously [34]. Briefly, the water maze consists of a circular tank with a diameter of 120 cm and a circular platform with a diameter of 6 cm. The circular tank was filled with room-temperature opaque water 60 cm deep, and the platform was immersed 1 cm below the water surface. Different shapes were pasted along the tank wall as spatial reference points. Above the maze, a digital tracking device was installed to record swimming tracks in the water maze. The mice were tested at the same time each day. During the incubation period, 6 consecutive days of underwater platform training were conducted, with each phase consisting of four tests. In each test, the animals were released from the tank walls and were allowed to search for and stand on the hidden platform during the 60-s test. If the animals could not find the platform within the specified time, they were guided to the platform and allowed to stay on the platform for 15 s, which was convenient for learning and directional memory. Probe testing was performed 24 h after training. In the probe test, the platform was removed, and mouse performance was recorded for 60 s. The time spent in the target quadrant, the delay to reach the platform, the distance to reach the platform, and the number of platforms were automatically recorded for subsequent analysis.

### Novel object recognition

The novel object recognition test was carried out as described previously [14]. Briefly, the mice were placed in the laboratory and allowed to adapt for 30 min for 2 consecutive days before the test. On the first day, the mice were placed in an open field, and two identical substances (familiar substances) appeared on the side of the empty box to allow free exploration of the object for 5 min of the device (50 × 50 × 50 cm). Then, after an hour, a familiar substance was substituted with a new substance, and the mice were allowed to probe for another 5 min. The time spent exploring the two substances was recorded using a digital video tracking system (Zhongshidichuang, Beijing, China). The results are indicated as touching the substance (tail only excluded) or facing the substance (distance < 2 cm). To explore CA1-dependent cognition, we analysed the preference rate of time spent on the novel substance to the total exploration time and demonstrated that the preference for the novel substance was higher in total spatial recognition memory.

### Statistical analysis

GraphPad Prism (version 8.0.1, GraphPad Software Inc.) was applied for the statistical analysis. All values are presented as the mean ± SEM. The unpaired two-tailed Student’s t test was used to analyse differences between groups (only 2 groups). Multiple groups (> 2 groups) were compared by one-way or two-way ANOVA followed by Dunnett’s or Tukey’s post hoc test. In the MWM test, repeated-measures (RM) ANOVA was used to analyse the training phase (6 days). The nonparametric Kruskal–Wallis rank-sum test was used to analyse nonnormal distributions. A *P value* < 0.05 was considered statistically significant.

## Results

### SIRT5 desuccinylates lysine 166 of ANXA1 after cerebral ischaemia

**Fig. 1:**
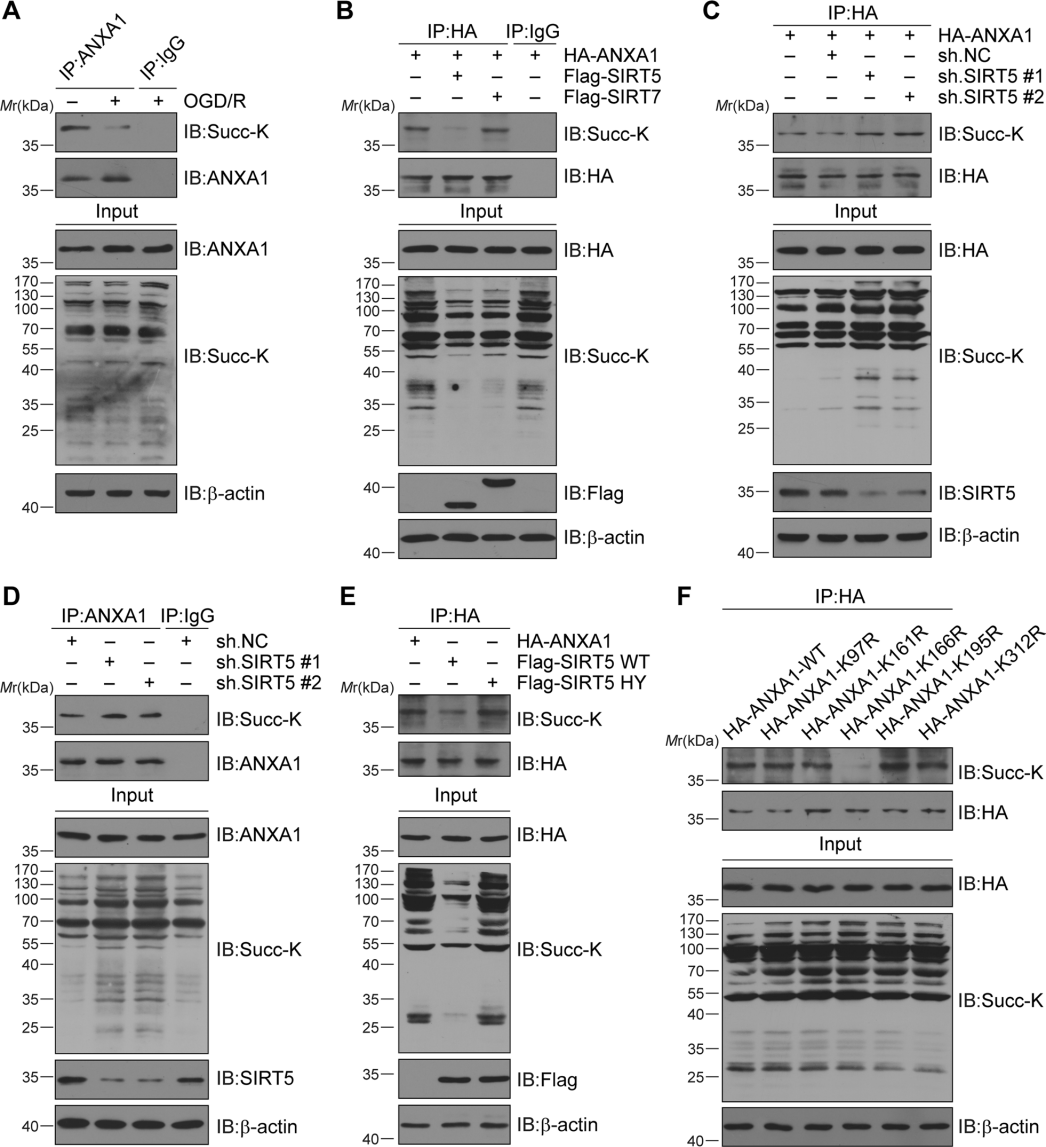
SIRT5 binds to and desuccinylates ANXA1 at K166 after OGD/R. **A** Primary cultured microglia were subjected to oxygen and glucose deprivation/reoxygenation (OGD/R) for 24 h. Coimmunoprecipitation (Co-IP) analysis indicating the succinylation level of ANXA1. **B** HEK293T cells were transfected with plasmids encoding Flag-tagged SIRT5 and SIRT7 together with haemagglutinin (HA)-tagged ANXA1. Co-IP analysis showing the succinylation level of ANXA1. **C** HEK293T cells were transfected with HA-ANXA1 and subjected to SIRT5 knockdown. Co-IP analysis showing the succinylation level of ANXA1. **D** Co-IP analysis showing the succinylation level of ANXA1 in primary cultured microglial cells subjected to SIRT5 knockdown. **E** HEK293T cells were transfected with plasmids encoding HA-ANXA1 and Flag-tagged wild-type SIRT5 (SIRT5-WT) or SIRT-H158Y (SIRT5-HY). Co-IP analysis was conducted to show the succinylation level of ANXA1. **F** HEK293T cells were transfected with plasmids encoding HA-ANXA1-WT, K97R, K161R, K166R, K195R, or K312R mutants, respectively. The cell extracts were collected for IP with anti-HA beads, followed by IB analysis with the indicated antibodies and showing the succinylation level of ANXA1. Data represent at least three independent experiments. *M*_r_, relative molecular mass

We first examined whether ANXA1 could be modified by succinylation in microglia. To address this question, we conducted a coimmunoprecipitation (Co-IP) assay to study the succinylation level of ANXA1. The results demonstrated that ANXA1 could be modified by succinylation, and its succinylation level was decreased after OGD/R (Fig. 1A). Given that protein desuccinylation is mainly regulated by SIRT5 or SIRT7 [24], we investigated which enzyme mediates the desuccinylation of ANXA1. The data showed that overexpression of SIRT5, but not SIRT7, markedly decreased the succinylation level of ANXA1 (Fig. 1B). To further confirm that SIRT5 functions as a desuccinylase of ANXA1, knockdown of endogenous SIRT5 by two specific short hairpin RNAs (sh-SIRT5#1 or sh-SIRT5#2) increased both ectopically expressed and endogenous ANXA1 succinylation levels in HEK293T cells and primary microglia, respectively (Fig. 1C, D). Then, HEK293T cells were transfected with a construct expressing wild-type (SIRT5-WT) or a catalytic-deficient mutant SIRT5 [35] (SIRT5-H158Y, histidine-to-tyrosine mutation at residue 158). Compared to SIRT5-WT, overexpression of SIRT5-HY (SIRT5-H158Y) failed to deconjugate succinyl-CoA from ANXA1 (Fig. 1E). In addition, MC3482, a SIRT5 specific inhibitor, could significantly reversed the succinylation levels of ANXA1 after OGD/R (Additional file 1: Fig. S1). To identify the specific K_succ_ sites in ANXA1, bioinformatic software algorithms (http://systbio.cau.edu.cn/SuccinSite/) [36] were utilized, and five potential sites (K97, K161, K166, K195, K312) were identified. Next, we mutated each of these five lysines to arginine (R; conserves the positive charge, mimicking the desuccinylated state) and examined their succinylation levels. As shown in Fig. 1F, only K166R mutant exhibited lower succinylation levels, demonstrating that K166 in ANXA1 may be the key succinylation site. Moreover, we found that lysine 166 of ANXA1 is evolutionarily conserved across species (Additional file 3: Fig. S2). Altogether, these data suggest that ANXA1 could be modified by succinylation and that SIRT5 desuccinylates ANXA1 at lysine 166.

### SIRT5 expression increases in response to ischaemic stroke

**Fig. 2:**
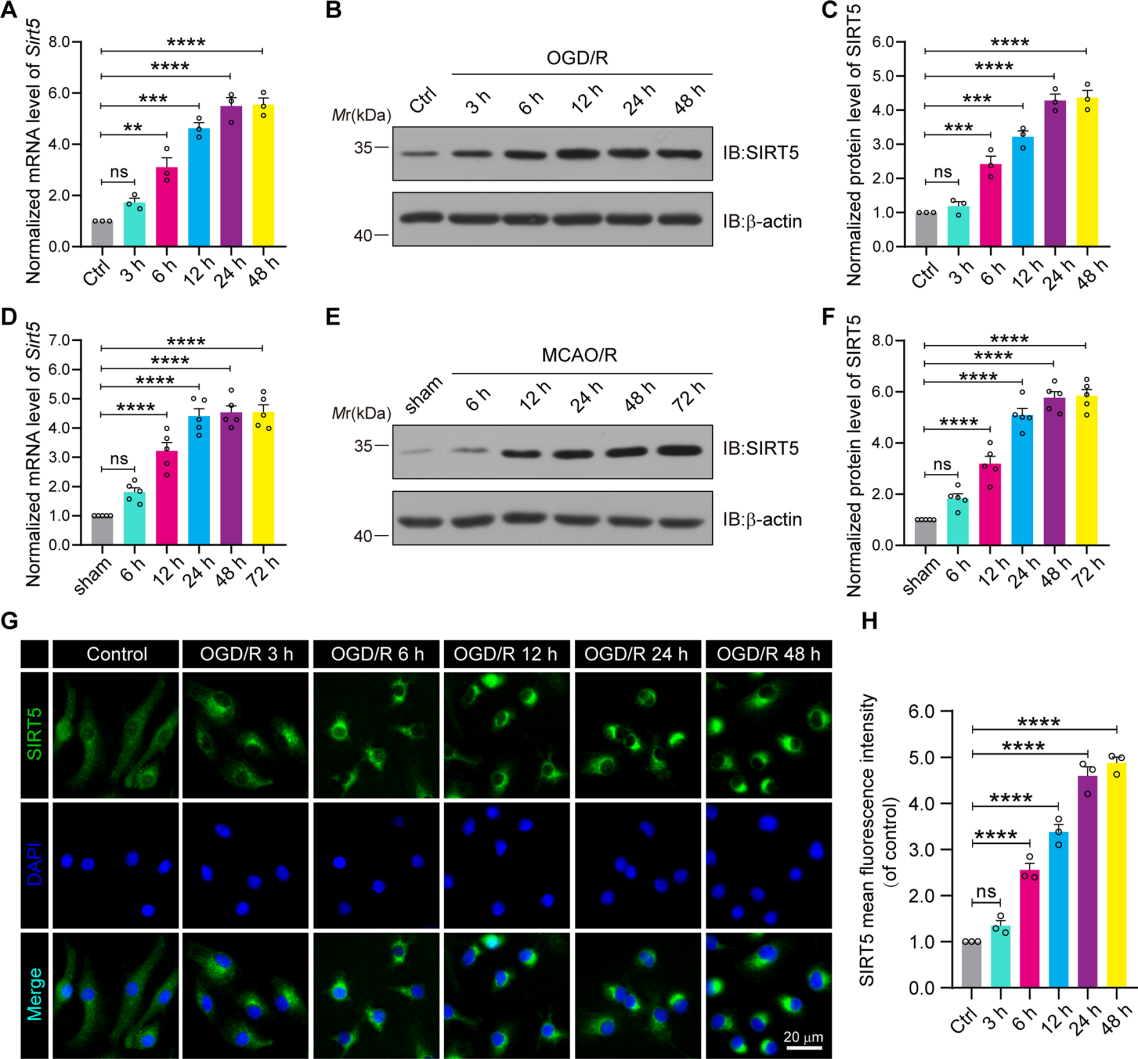
SIRT5 is upregulated after cerebral I/R injury. **A** qPCR assay indicating *Sirt5* mRNA expression in primary cultured microglia at the indicated time points following OGD. *n* = 3 per time point. **B**, **C** Primary cultured microglia were subjected to OGD and reoxygenation at the indicated time points. Western blot assay (**B**) and quantification analysis (**C**) showing SIRT5 protein expression. *n* = 3 per time point. **D** qPCR assay indicating *Sirt5* mRNA expression in cerebral tissues at the indicated time points following cerebral ischaemia injury. *n* = 5 per time point. **E**, **F** Mouse brain homogenates were extracted after MCAO surgery for 1 h and reperfusion at the indicated time points. Western blot assay (**E**) and quantification analysis (**F**) showing SIRT5 protein expression; *n* = 5 per time point. **G** Primary cultured microglia were subjected to OGD and reoxygenation at the indicated time points. Immunofluorescence assays of SIRT5 (green) with colabelled 4′,6-diamidino-2-phenylindole (DAPI; blue, nuclei). Scale bars, 20 μm. **H** The fluorescence intensity of SIRT5 was quantified using ImageJ software. Data are presented as the mean ± SEM. ns: no significance, **P < 0.01, ***P < 0.001, and ****P < 0.0001

To assess the function of SIRT5 in ischaemic stroke, we explored the expression level of SIRT5 in vivo and in vitro. First, in primary microglia subjected to OGD and reoxygenation at several different time points, qPCR analysis showed that the level of *Sirt5* mRNA increased at 3 h and peaked at 24 h after OGD (Fig. 2A). Meanwhile, western blot also showed that SIRT5 protein levels gradually increased after OGD/R (Fig. 2B, C). Next, the SIRT5 level was further investigated in vivo. An experimental ischaemic stroke animal model was established by MCAO surgery for 1 h followed by various periods of reperfusion from 6 to 72 h. Consistent with the in vitro observations, qPCR analysis and western blot results also revealed that the mRNA and protein levels of SIRT5 were progressively increased with longer periods of I/R (Fig. 2D–F). Furthermore, we performed experiments to explore the expression level of SIRT5 in the contralateral hemisphere and ipsilateral hemisphere (ischaemic hemisphere) of the mice underwent MCAO/R surgery. As shown in the Additional file 3: Fig. S3, qPCR analysis and western blot results collectively indicate that SIRT5 mRNA and protein expression was significantly increased in the ipsilateral hemisphere after cerebral I/R injury, as compared to the contralateral hemisphere and sham controls. Finally, immunofluorescence analysis also showed that the SIRT5-positive fluorescent signals gradually increased in primary cultured microglia after OGD/R treatment (Fig. 2G, H and Additional file 4: Fig. S4B). Taken together, our data suggested that cerebral I/R injury induced the expression of SIRT5.

### OGD/R increases the binding of ANXA1 with SIRT5

**Fig. 3:**
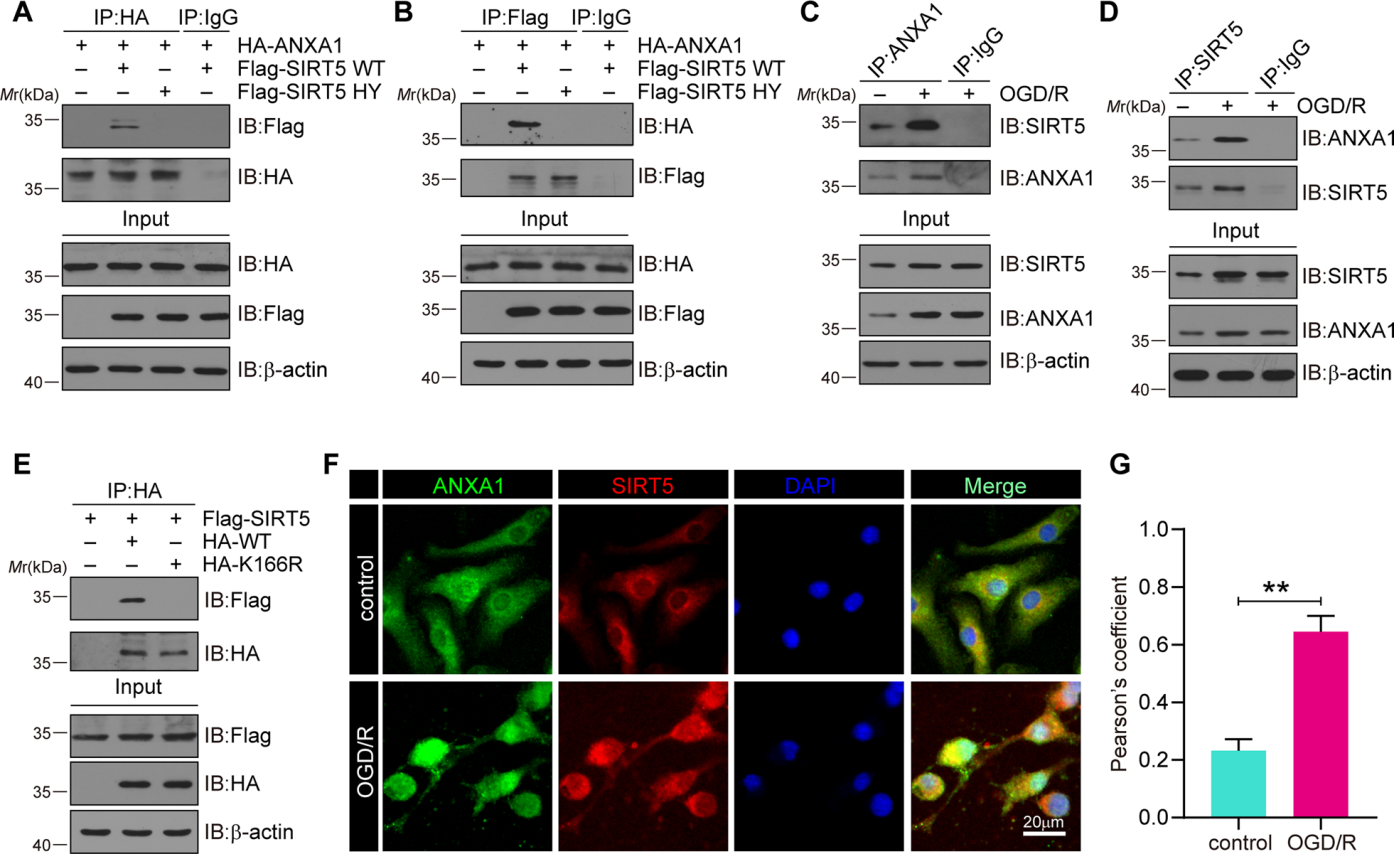
The interaction of SIRT5 and ANXA1 increases after OGD/R. **A**, **B** HEK293T cells were transfected with plasmids encoding Flag-SIRT5-WT or Flag-SIRT5-H158Y together with HA-ANXA1. The cell extracts were collected for IP with anti-HA (**A**) or anti-Flag beads (**B**), followed by IB analysis with the indicated antibodies and showing the interaction of ectopically expressed ANXA1 and SIRT5. **C**, **D** Primary cultured microglia were subjected to OGD/R. Co-IP analysis indicating the interaction of endogenous ANXA1 and SIRT5. **E** HEK293T cells were transfected with plasmids encoding HA-ANXA1-WT or HA-ANXA1-K166R together with Flag-SIRT5. The cell lysates were collected for IP with anti-HA beads, followed by IB analysis with the indicated antibodies, showing the interaction of ectopically expressed ANXA1 and SIRT5. **F** Primary cultured microglia were subjected to OGD/R. Immunofluorescence assays of ANXA (green) and SIRT5 (red) with colabelled DAPI (blue, nuclei). Scale bars, 20 μm. **G** Quantitative analysis of the colocalization was conducted by Pearson’s coefficient measurement using ImageJ software. Data are presented as the mean ± SEM from at least three independent experiments. **P < 0.01

Next, to determine whether ANXA1 is a putative SIRT5 substrate, we first explored the interaction of ANXA1 and SIRT5. HEK293T cells were transfected with haemagglutinin (HA)-tagged ANXA1-WT and Flag-tagged SIRT5-WT or SIRT5-HY expression plasmids. We found that ectopically expressed ANXA1 could interact with SIRT5-WT but not with SIRT5-HY (Fig. 3A). This result was also confirmed by reverse co-IP assays (Fig. 3B). Furthermore, the binding of endogenous SIRT5 and ANXA1 in primary microglia was confirmed. The results revealed that OGD/R treatment dramatically increased the ANXA1-SIRT5 interaction (Fig. 3C, D). In addition, HEK293T cells were transfected with Flag-SIRT5-WT and HA-ANXA1-WT or HA-ANXA1-K166R expression plasmids. The results showed that SIRT5 could bind to ANXA1-WT rather than to ANXA1-K166R (Fig. 3E). Finally, we examined whether SIRT5 colocalized with ANXA1 by immunofluorescence staining in primary cultured microglia. As expected, the data also revealed that the colocalization of SIRT5 and ANXA1 increased after OGD/R (Fig. 3F, G). Collectively, these data concluded that the interaction of ANXA1 with SIRT5 was enhanced following cerebral I/R injury.

### Desuccinylation of ANXA1 by SIRT5 promotes ANXA1 nuclear localization and decreases ANXA1 membrane recruitment

**Fig. 4:**
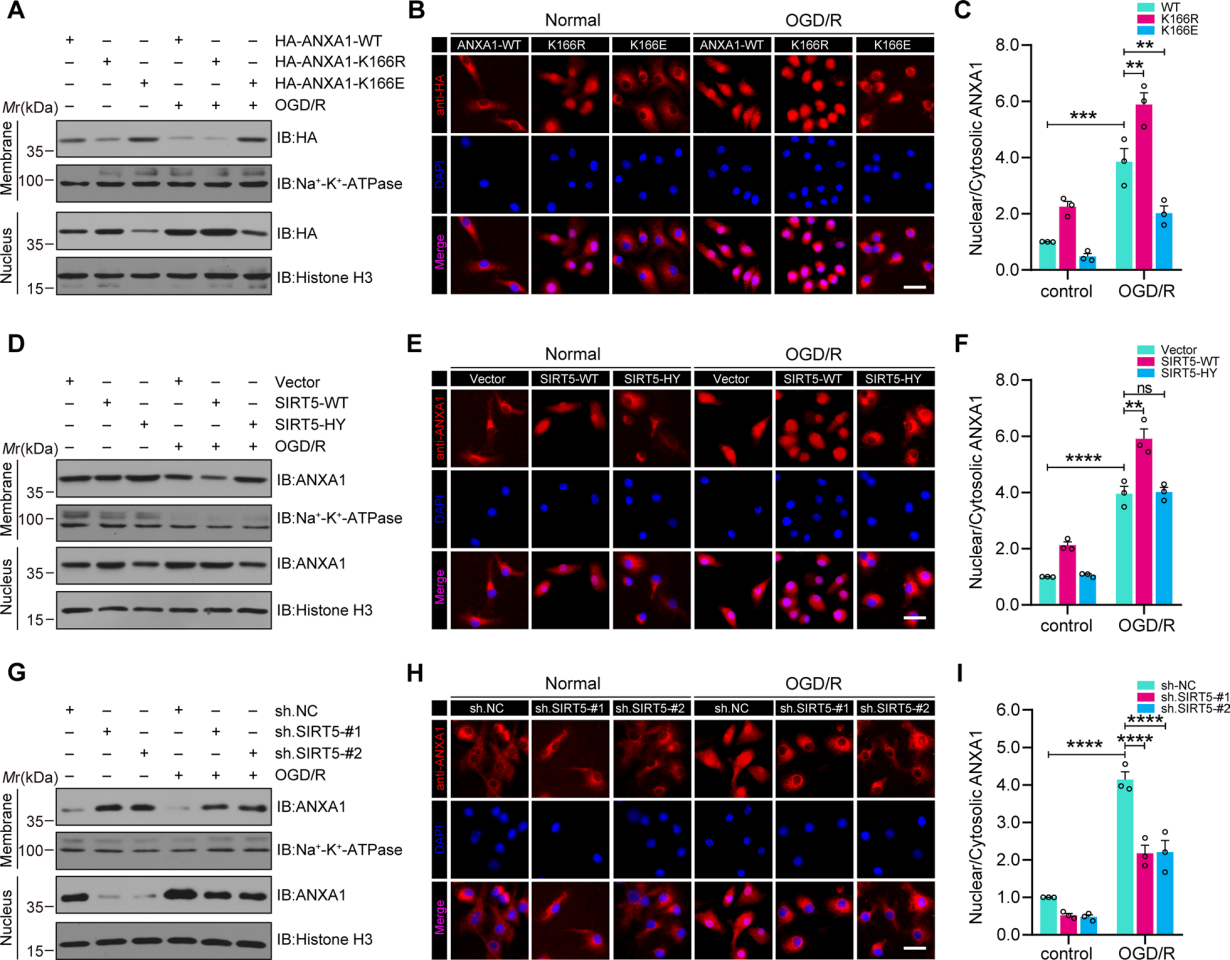
SIRT5 regulates the subcellular localization of ANXA1. **A** Primary cultured microglia were infected with adenovirus encoding HA-ANXA1-WT, HA-ANXA1-166R or HA-ANXA1-166E and subjected to OGD/R. Western blot assay indicating ANXA1 levels in membrane and nuclear fractions. **B** Immunofluorescence assays of HA-ANXA1 (red) with colabelled DAPI (blue, nuclei), showing the subcellular location of HA-ANXA1. **C** Quantitative analysis of nuclear/cytoplasmic HA-ANXA1 levels in (**B**). **D** Primary cultured microglia were infected with adenoviruses encoding Flag-SIRT5-WT and Flag-SIRT5-H158Y and subjected to OGD/R. Western blot assay indicating ANXA1 levels in membrane and nuclear fractions. **E** Immunofluorescence assays of ANXA1 (red) with colabelled DAPI (blue, nuclei), showing the subcellular location of ANXA1. **F** Quantitative analysis of nuclear/cytoplasmic ANXA1 levels in (**E**). **G** Primary cultured microglia were infected with adenovirus encoding the SIRT5 shRNA sequence and subjected to OGD/R. Western blot assay indicating ANXA1 levels in membrane and nuclear fractions. **H** Immunofluorescence assays of ANXA1 (red) with colabelled DAPI (blue, nuclei), showing the subcellular location of ANXA1. **I** Quantitative analysis of nuclear/cytoplasmic ANXA1 levels in (**H**). Scale bars, 20 μm. Data are presented as the mean ± SEM from at least three independent experiments. ns: no significance, **P < 0.01, ***P < 0.001, and ****P < 0.0001

Based on the aforementioned results, the role of ANXA1 in neuroinflammation relies on its subcellular localization. Succinylation accommodates the translocation of many proteins between the cytoplasm and nucleus [37], and we suspected that the SIRT5-mediated desuccinylation of ANXA1 would regulate its subcellular localization. To this end, primary cultured microglia were first infected with adenovirus encoding HA-ANXA1-WT, HA-ANXA1-166R or HA-ANXA1-166E (E, mimics lysine succinylation), respectively. The nuclear and membrane fractions were extracted, and western blot results indicated that OGD/R increased ANXA1-WT nuclear accumulation and decreased its membrane accumulation, whereas the nuclear accumulation of ANXA1-K166R further increased and the membrane accumulation of ANXA1-K166R further decreased compared to those of ANXA1-WT overexpression groups. Meanwhile, we found that ANXA1-K166E nuclear accumulation markedly decreased, and its membrane accumulation further increased compared to the ANXA1-WT overexpression groups (Fig. 4A). Similar results were also confirmed by immunofluorescence assay. The results showed that the ANXA1-K166R mutant was primarily localized in the nuclear fragment. In contrast to ANXA1-WT, the ANXA1-K166E mutant primarily localized to cytoplasmic fragments (Fig. 4B, C). To further verify the influence of SIRT5-mediated ANXA1 desuccinylation on its subcellular translocation, primary microglia were infected with adenovirus encoding Flag-SIRT5-WT or the Flag-SIRT5-HY mutant. The nuclear/cytoplasm fractionation experiment results indicated that SIRT5-WT increased ANXA1 nuclear accumulation and decreased its membrane recruitment, whereas SIRT5-HY had little effect on the subcellular localization of ANXA1 (Fig. 4D). Similar results were also observed by immunofluorescence staining (Fig. 4E, F). Last, SIRT5 expression in primary microglia was knocked down by specific shRNAs. Western blot results showed that SIRT5 inhibition obviously lessened ANXA1 nuclear translocation induced by OGD/R and increased its membrane accumulation (Fig. 4G). Similar results were also confirmed by immunofluorescence staining (Fig. 4H, I). Based on these results, SIRT5 was determined to function as a positive regulator to increase ANXA1 nuclear accumulation and decrease ANXA1 membrane recruitment after OGD/R.

### SIRT5 decreases ANXA1 secretion and its binding with FPR2 after OGD/R injury

**Fig. 5:**
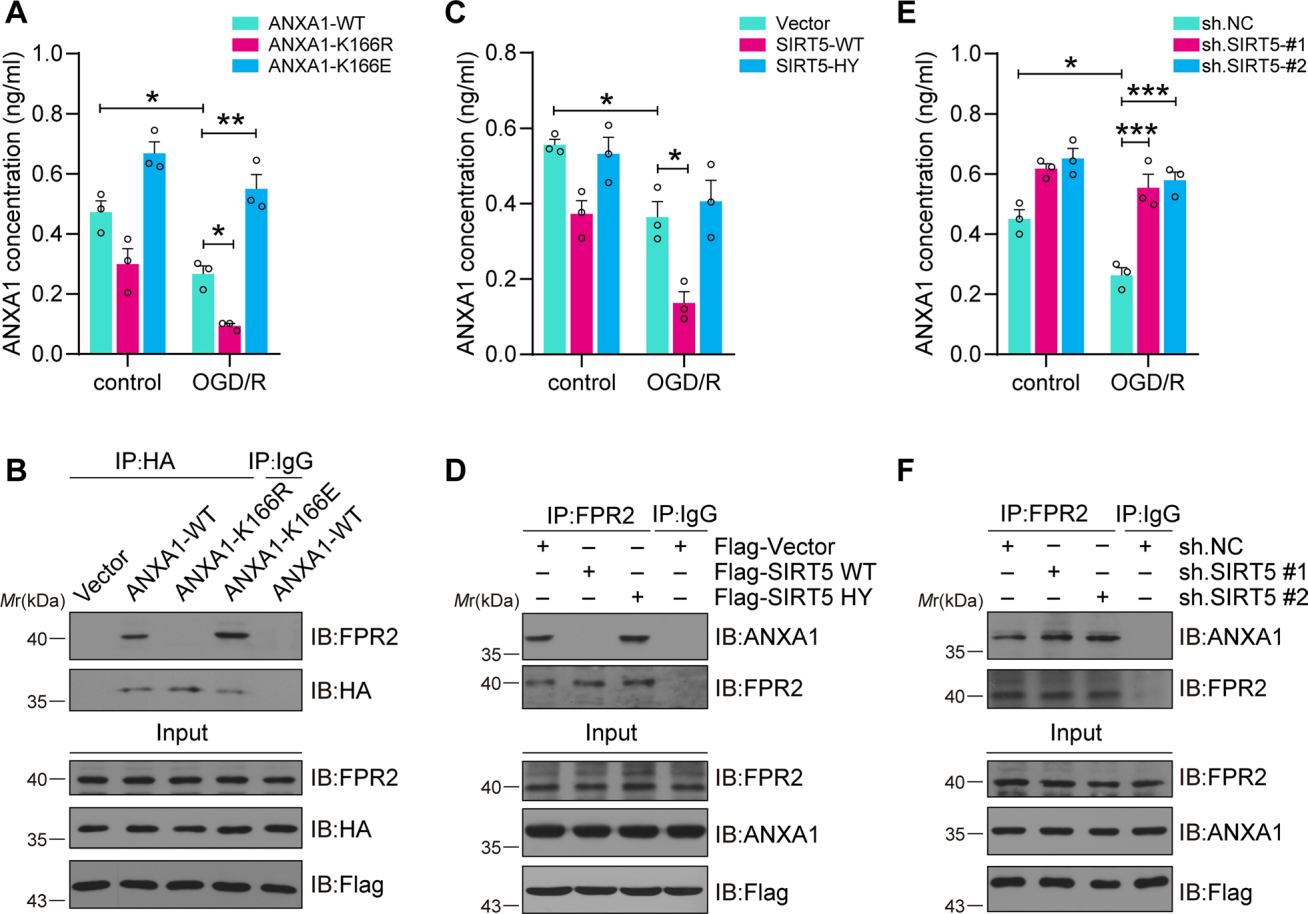
SIRT5 suppresses ANXA1 secretion after OGD/R. **A**, **B** Primary cultured microglia were infected with adenovirus encoding HA-ANXA1-WT, HA-ANXA1-166R or HA-ANXA1-166E and subjected to OGD/R. ELISA analysis indicating the secretion level of ANXA1 (**A**). Co-IP assays showed the binding of ectopically expressed ANXA1 with endogenous FPR2 (**B**). **C**, **D** Primary cultured microglia were infected with adenoviruses encoding Flag-SIRT5-WT and Flag-SIRT5-H158Y and subjected to OGD/R. ELISA analysis indicating the secretion level of ANXA1 (**C**). Co-IP assays showed the binding of endogenous ANXA1 with FPR2 (**D**). **E**, **F** Primary cultured microglia were infected with adenovirus encoding SIRT5 shRNA sequence #1 or #2 and subjected to OGD/R. ELISA analysis indicating the secretion level of ANXA1 (**E**). Co-IP assays showing the binding of endogenous ANXA1 with FPR2 (**F**). Data are presented as the mean ± SEM from at least three independent experiments. ns: no significance, *P < 0.05, **P < 0.01, ***P < 0.001

A previous study showed that ANXA1 transport to the cell membrane and interaction with FPR2 are involved in its anti-inflammatory effect [38]. After identifying the reduction in ANXA1 membrane accumulation caused by SIRT5, we then sought to ascertain the level of ANXA1 secretion and its binding with FPR2. Frist, primary cultured microglial cells were transduced with adenovirus encoding HA-ANXA1-WT/-K166R/-K166E and then treated with or without OGD/R. The ELISA results demonstrated that the secretion amount of ANXA1 obviously decreased after ANXA1-K166R transfection in contrast to ANXA1-WT, but increased after ANXA1-K166E transfection under OGD/R conditions (Fig. 5A). Meanwhile, the co-IP results indicated that the ANXA1-K166R mutant presented decreased binding to FPR2, as compared with ANXA1-WT, while ANXA1-K166E mutant showed increased binding to FPR2 (Fig. 5B). To further verify the influence of SIRT5-mediated ANXA1 desuccinylation on ANXA1 secretion, primary microglial cells were transduced with adenovirus encoding Flag-SIRT5-WT/-HY and treated with OGD/R. ELISA results indicated that SIRT5-WT decreased the level of ANXA1 secretion, but SIRT5-HY showed little effect (Fig. 5C). In addition, the co-IP results also suggested that SIRT5-WT blocked the binding of ANXA1 and FPR2, while SIRT5-HY showed little effect, as compared with vector group (Fig. 5D). Last, SIRT5 in primary microglial cells was knocked down by two specific shRNAs. The ELISA results uncovered that knockdown of SIRT5 increased the secretion level of ANXA1 after OGD/R treatment (Fig. 5E). Moreover, co-IP assays indicated that the combination of endogenous ANXA1 and FPR2 notably increased after SIRT5 had been knockdown (Fig. 5F). Collectively, these data confirmed that SIRT5-mediated desuccinylation of ANXA1 could decrease ANXA1 secretion and its binding with FPR2 during OGD/R.

### SIRT5-mediated desuccinylation of ANXA1 decreases its SUMOylation level

**Fig. 6:**
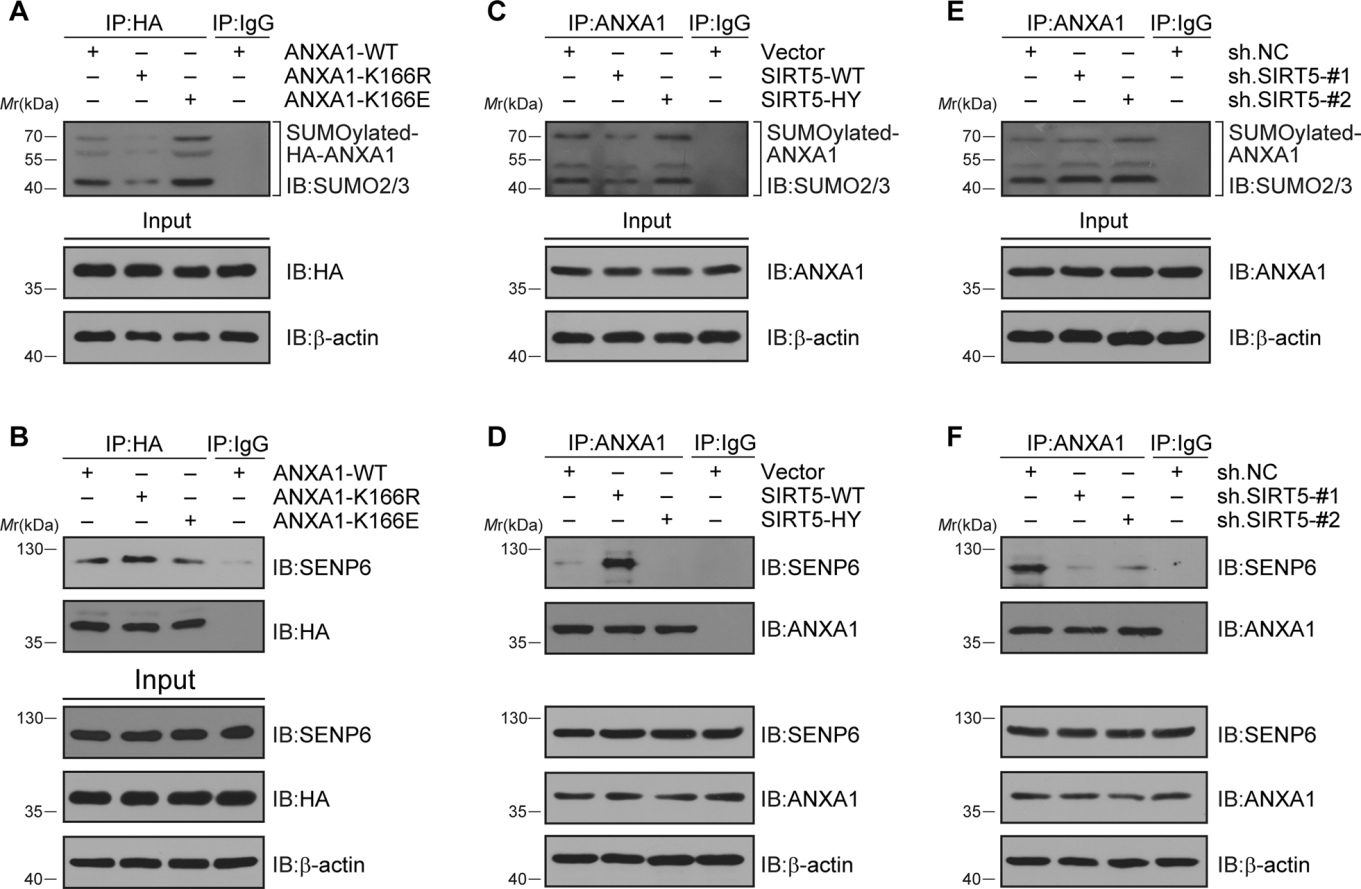
SIRT5-induced desuccinylation of ANXA1 decreased its SUMOylation level. **A**, **B** Primary cultured microglia were infected with adenovirus encoding HA-ANXA1-WT, HA-ANXA1-166R or HA-ANXA1-166E. A co-IP assay was performed to confirm the SUMOylation levels of ANXA1 (**A**) and the interaction of ectopically expressed ANXA1 with SENP6 (**B**). **C**, **D** Primary cultured microglia were infected with adenovirus encoding SIRT5-WT or SIRT5-HY. Co-IP confirmed the SUMOylation levels of ANXA1 (**C**) and the interaction of endogenous ANXA1 with SENP6 (**D**). **E**, **F** Primary cultured microglia were infected with adenovirus encoding shRNAs against SIRT5 or nontargeted scramble control. A co-IP assay was performed to confirm the SUMOylation levels of ANXA1 (**E**) and the interaction of endogenous ANXA1 with SENP6 (**F**). Data represent at least three independent experiments

Our recent study showed that SENP6-mediated deSUMOylation is critical for ANXA1 subcellular localization [18]. To reveal the mechanisms underlying SIRT5-induced ANXA1 nuclear translocation, we investigated the crosstalk between ANXA1 succinylation and SUMOylation. First, primary cultured microglia were transduced with adenoviruses encoding HA-ANXA1-WT/-K166R/-K166E and challenged with OGD/R treatment. The co-IP results revealed that the SUMOylation level of ANXA1 obviously decreased after ANXA1-K166R, while increased after ANXA1-K166E in contrast to ANXA1-WT transfection (Fig. 6A). In addition, the interaction between SENP6 and ANXA1 was significantly enhanced after ANXA1-K166R transfection (Fig. 6B). Next, adenoviruses encoding Flag-SIRT5-WT/-HY were used to infect microglial cells. The co-IP results suggested that SIRT5-WT dramatically decreased the SUMOylation level of ANXA1, while SIRT5-HY had little effect (Fig. 6C). Furthermore, compared to vector and SIRT5-HY transfection, SIRT5-WT transfection also obviously increased the interaction of SENP6 with ANXA1 in microglial cells (Fig. 6D). Finally, we validated that knockdown of SIRT5 by two independent shRNAs in primary microglial cells markedly increased the SUMOylation level of ANXA1 after OGD/R (Fig. 6E). The co-IP results also showed that shRNA-mediated SIRT5 knockdown notably decreased the interaction of SENP6 with ANXA1 in microglial cells (Fig. 6F). Collectively, these data suggest that SIRT5-mediated desuccinylation of ANXA1 could promote the interaction of SENP6 with ANXA1 and decrease the SUMOylation level of ANXA1.

### SIRT5 aggravates microglia-induced neuroinflammation during OGD/R

**Fig. 7:**
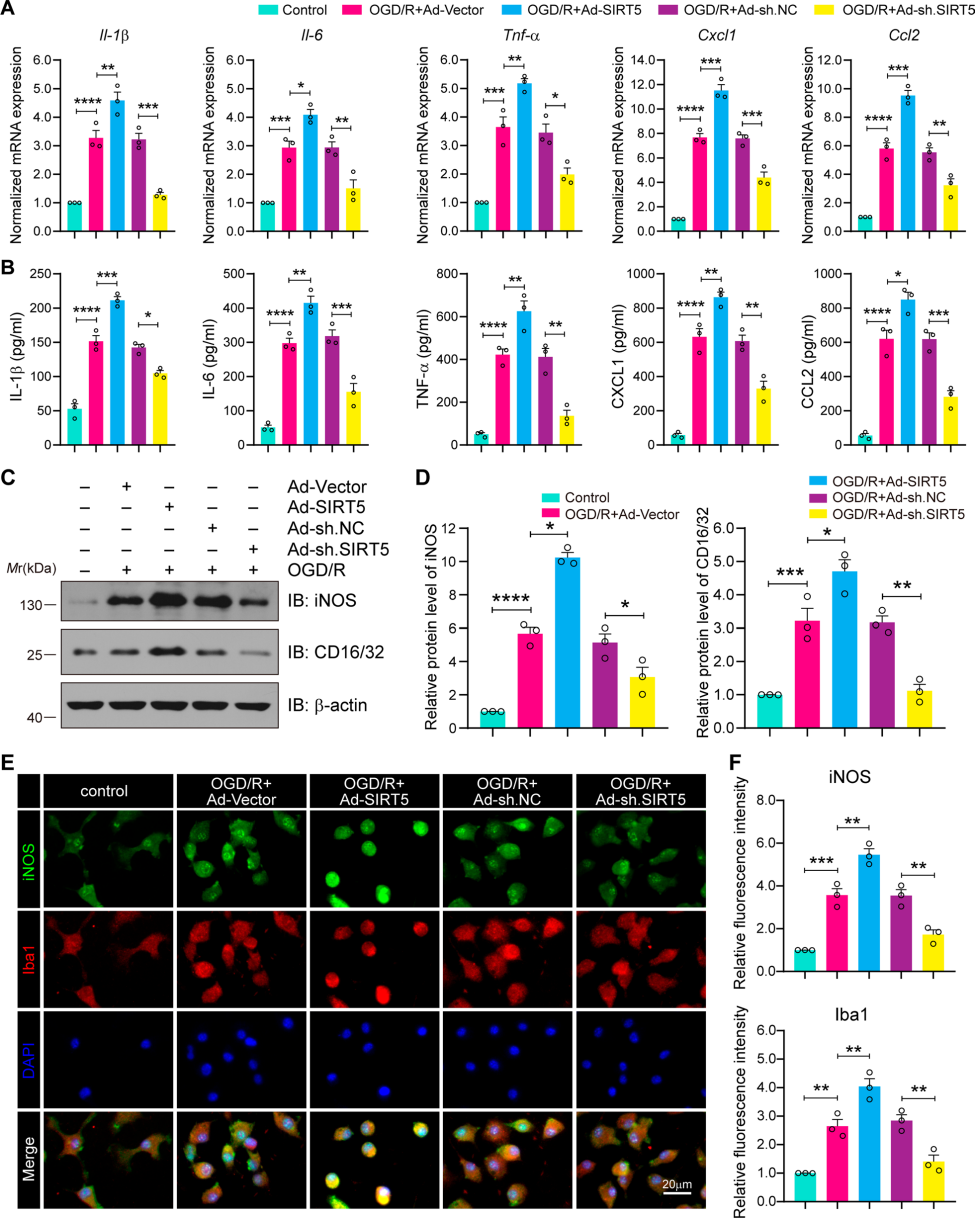
SIRT5 promotes OGD/R-induced expression of proinflammatory cytokines and chemokines in microglia. **A** Primary microglial cells were transduced with recombinant adenovirus carrying the SIRT5 coding sequence or the shRNA sequence. qPCR assay indicating the mRNA expression levels of *Il-1β*, *Il-6*, *Tnf-α*, *Cxcl1* and *Ccl2* in primary cultured microglia from the indicated groups. **B** ELISA analysis showing the protein secretion levels of IL-β, IL-6, TNF-α, Cxcl1 and Ccl2 in primary cultured microglial cell supernatants from the indicated groups. **C**, **D** Western blot assay (**C**) and quantification analysis (**D**) indicating the protein expression levels of iNOS and CD16/32 in primary cultured microglia. **E** Immunofluorescence assays of iNOS (green) and Iba-1 (red) with colabelled DAPI (blue, nuclei). Scale bars, 20 μm. **F** The fluorescence intensity of iNOS and Iba-1 was quantified using ImageJ software. Data are presented as the mean ± SEM from at least three independent experiments. *P < 0.05, **P < 0.01, ***P < 0.001, and ****P < 0.0001

Given that ANXA1 plays a crucial role in microglia-induced inflammatory processes, we then attempted to investigate the biological significance of ANXA1 desuccinylation in microglia-induced inflammation after cerebral I/R injury. For this purpose, primary cultured microglia were transduced with recombinant adenovirus carrying the SIRT5 coding sequence or shRNA sequence. qPCR assay results confirmed that SIRT5 overexpression markedly upregulated the mRNA expression of proinflammatory cytokines and chemokines, such as *Il-1β*, *Il-6*, *Tnf-α*, *Cxcl1* and *Ccl2*. In contrast, SIRT5 knockdown exhibited the reverse effect (Fig. 7A). Meanwhile, the ELISA results also indicated that the secretion levels of the aforementioned proinflammatory factors were consistent with the mRNA expression (Fig. 7B). These findings were further verified by western blot to investigate the expression of the proinflammatory mediators iNOS (inducible nitric oxide synthase) and CD16/32 (Fig. 7C, D). Finally, immunofluorescence assay was performed to determine the levels of iNOS and Iba-1 (known as activated microglia). We found that OGD/R significantly increased the fluorescence intensity of iNOS and Iba-1 in primary cultured microglia. SIRT5 upregulation further increased the expression of iNOS and Iba-1, while SIRT5 inhibition exhibited the reverse effect (Fig. 7E, F). Collectively, these results indicate that SIRT5 aggravates the microglia-induced inflammatory response under OGD/R conditions.

### SIRT5 knockdown alleviates damage to neuronal cells co-cultured with proinflammatory microglia

**Fig. 8:**
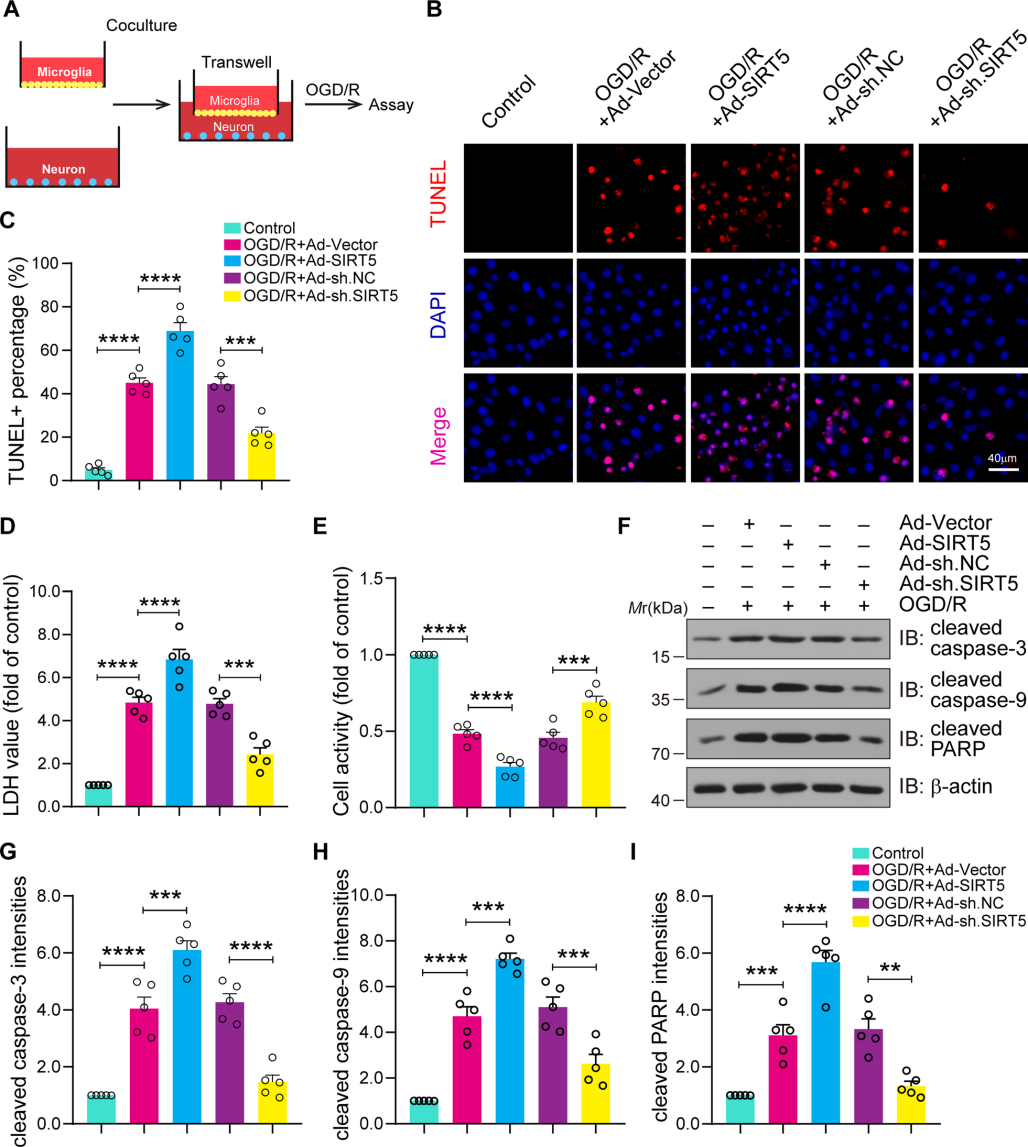
SIRT5 knockdown in microglia protects against OGD/R-induced neuronal damage. **A** Primary microglial cells were transduced with recombinant adenovirus carrying the SIRT5 coding sequence or shRNA sequence for 48 h and co-cultured with neurons via a Transwell system, followed by OGD/R treatment. Schematic representation as above. **B**, **C** Neuronal apoptosis was determined by TUNEL staining (**B**), and quantitative analysis was conducted to show the percentage of TUNEL^+^ neurons (**C**). **D** LDH release showing neuronal damage. **E** CCK-8 assay indicating neuronal viability. **F**–**I** Western blot assays (**F**) and quantification analysis showing the protein levels of cleaved caspase-3 (**G**), cleaved caspase-9 (**H**), and cleaved PARP (**I**) in neurons. Data are presented as the mean ± SEM from five independent experiments. **P < 0.01, ***P < 0.001 and ****P < 0.0001

We conclude that SIRT5 increases the expression of proinflammatory factors in primary cultured microglia after OGD/R (Fig. 7). We further investigated the role of SIRT5 in OGD/R-induced neuronal damage, and a microglia–neuron co-culture system was applied (Fig. 8A). Primary microglial cells were transduced with recombinant adenovirus carrying the SIRT5 coding sequence or shRNA sequence. Frist, the overexpression and knockdown efficiency of these adenovirus are presented in Additional file 5: Fig. S5, showing successful SIRT5 overexpression or knockdown in primary cultured microglial cells. Then, TUNEL staining indicated that the overexpression of SIRT5 obviously increased neuronal death induced by OGD/R. In contrast, SIRT5 inhibition in microglia significantly decreased neuronal death (Fig. 8B, C). Consistent with these results, the overexpression of SIRT5 in microglia obviously increased LDH release, but SIRT5 knockdown decreased LDH release (Fig. 8D). We used CCK-8 assays to assess neuronal viability. The results revealed that the overexpression of SIRT5 significantly decreased neuronal viability, but SIRT5 knockdown enhanced neuronal viability after OGD/R injury (Fig. 8E). Immunoblots also showed that the overexpression of SIRT5 in microglia significantly increased the expression levels of proapoptotic molecules, such as cleaved caspase-3, cleaved caspase-9 and cleaved PARP in neurons but exhibited the opposite trend in SIRT5 knockdown groups (Fig. 8F–I). These findings suggest that SIRT5 inhibition in microglia attenuates proinflammatory microglial-potentiated neurotoxic effects on ischaemic neurons.

### SIRT5 knockdown in microglia protects against cerebral I/R injury in a mouse MCAO model

**Fig. 9:**
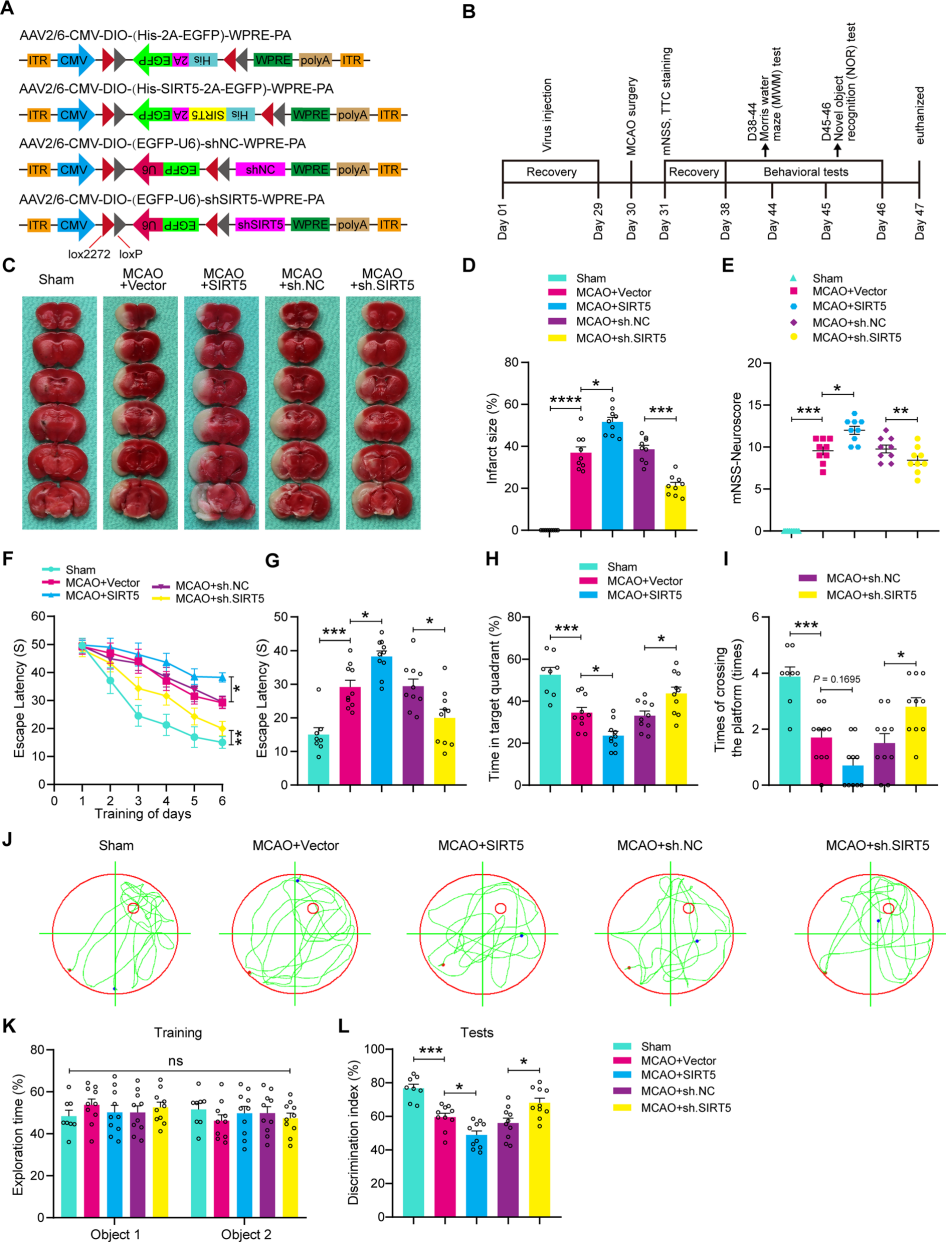
SIRT5 knockdown in microglia is neuroprotective during cerebral I/R injury. **A** Experimental design for microglia-specific overexpression and knockdown of SIRT5. AAV2/6 vectors encoding CMV-DIO-His-SIRT5 or CMV-DIO-EGFP-U6-shSIRT5 were stereotactically injected into the hippocampal CA1 region, cerebral cortex and striatum of Cx3cr1-Cre mice. **B** A diagram of the experimental process. **C**, **D** TTC staining indicating the infarction volume induced by ischaemic stroke (**C**) and quantification analysis showing the infarct size (**D**). *n* = 9 mice per group. **E** The modified neurological severity score (mNSS) showing the neurological deficit scores after ischaemic stroke. *n* = 9 mice per group. **F**–**J** Latency trial (**F**, **G**) and probe trial (**H**–**J**) tests in the Morris water maze (MWM). *n* = 8–10 mice per group. **F** The mean escape latency of the MWM tests during Days 1–6 of testing. **G** The mean escape latency to the hidden platform on Day 6 in the MWM tests. **H** Time (in seconds, s) spent in the target quadrant on Day 7. **I** T Number of times crossing the target platform location on Day 7. **J** Representative path tracing on Day 7 of the probe test. **K**, **L** Novel object recognition (NOR) testing. **K** The time spent exploring two identical objects of the mice in the familiarization trial. **L** The percentage of time spent exploring the novel and familiar objects in the test trial. *n* = 8–10 mice per group. Data are presented as the mean ± SEM. ns: no significance, *P < 0.05, **P < 0.01, ***P < 0.001 and ****P < 0.0001

Our in vitro studies showed that SIRT5 knockdown in microglia has protective effects against neuronal damage caused by microglial hyperactivation after OGD/R injury. Hence, we supposed that SIRT5 inhibition may protect against activated microglia-induced neurotoxicity in cerebral I/R injury in vivo. For this purpose, we applied a previously reported approach to specifically intervene in SIRT5 expression in brain microglial cells [14, 39, 40]. Accordingly, we constructed an adeno-associated virus type 2/6 (AAV2/6) carrying a SIRT5 shRNA sequence or SIRT5 coding sequence that only regulates the expression of SIRT5 in cells expressing Cre recombinase driven by the microglia-specific Cx3cr1 promoter (Fig. 9A). Each AAV vector was stereotactically injected into the cerebral cortex, hippocampal CA1 region, and striatum of adult Cx3cr1-Cre male mice. Four weeks after AAV injection, mice were subjected to MCAO surgery. Next, histological and behavioural studies were performed at the indicated time points (Fig. 9B). The infection efficiency of AAV-SIRT5 and AAV-sh.SIRT5 in brain tissue was confirmed by western blot, showing that the protein level of SIRT5 was significantly increased in mice-treated AAV-SIRT5. In addition, the results also revealed reduced endogenous SIRT5 expression in mice injected with AAV-sh.SIRT5, as compared to the sh.NC-treated mice, indicating successful SIRT5 knockdown in microglia (Additional file 6: Fig. S6).

We first investigated the brain infarct volume by 2,3,5-triphenyl-2H-tetrazolium chloride (TTC) staining after 24 h of reperfusion. The data showed that the SIRT5-overexpressing mice exhibited an increased cerebral infarct volume in contrast to the control mice, whereas the SIRT5 ablation mice showed a reduced cerebral infarct volume (Fig. 9C, D). Next, we calculated the modified neurological severity score (mNSS) to assess neurological deficits. The results showed that overexpression of SIRT5 enhanced neurological deficits, whereas SIRT5 knockdown alleviated neurological deficits (Fig. 9E). Then, we performed Morris water maze (MWM) testing to explore the effects of SIRT5 on spatial learning and memory functions. The data implied that SIRT5-overexpressing mice exhibited significant cognitive deficits, whereas SIRT5-knockdown mice displayed obvious improvements in cognitive performance, including the latency to reach the underwater platform, the time spent in the target quadrant, and the number of platform crossings (Fig. 9F–I).

We further explored the effect of SIRT5 on the cognitive function of the mice by the novel object recognition (NOR) task (Fig. 9K, L). The results revealed that the SIRT5-overexpressing group spent less time exploring the new object, while the SIRT5 knockdown group displayed a remarkable predilection for the new object, suggesting the improvement of declarative recognition memory. Based on the above results, we conclude that SIRT5 knockdown in microglia significantly decreased cerebral infarct volume, reduced neurological deficit scores, and protected cognitive function against cerebral I/R injury.

## Discussion

In the current study, we studied the impact of cerebral I/R injury-induced ANXA1 desuccinylation on microglial-induced neuroinflammation and the underlying mechanism. Specifically, we demonstrated that ANXA1 was succinylated at K166 and that SIRT5 mediated its desuccinylation. Moreover, we found that SIRT5 promoted the nuclear translocation of ANXA1 and decreased its membrane recruitment and extracellular secretion. Further work revealed that suppressing SIRT5 markedly decreased microglial-induced proinflammatory cytokines and chemokines production and alleviated OGD/R-induced neuronal damage. In addition, SIRT5 knockdown in specific microglia protected against I/R injury in a mouse MCAO model. Thus, SIRT5 might be a promising therapeutic target for the treatment of ischaemic stroke.

Succinylation is a relatively recently revealed novel PTM in which metabolically derived succinyl CoA modifies protein lysine groups, resulting in a protein flip from positive to negative and a relatively large increase in mass compared with other PTMs [22, 41]. A global lysine succinylation proteome analysis showed that ANXA1 in HeLa cells can be succinylated [19]. Consistent with this result, we provided experimental evidence that ANXA1 could be modified by succinylation and that lysine 166 was the specific K_succ_ site in ANXA1. Previous research has demonstrated that succinylation regulates behaviours during cytopathologic and physiological processes by altering the activity, stability, transport, and interaction with other molecules [42–44]. Wang et al. found that SIRT5-dependent desuccinylation of PKM2 can block its nuclear translocation [37]. In the current study, we demonstrated that ANXA1 succinylation also played a vital role in its subcellular localization. Interestingly, SIRT5-mediated desuccinylation of ANXA1 promotes its nuclear accumulation while decreasing its membrane recruitment and release after cerebral I/R injury.

Protein succinylation is regulated by sirtuins, members of the histone deacetylase family whose deacetylase activity is dependent on nicotinamide adenine dinucleotide (NAD^+^). Among members of the sirtuin family, SIRT5 has weak deacetylase activity, but it can catalyse the removal of three acidic lysine modifications, malonylation, succinylation, and glutarylation [45–47]. Previous studies have shown that SIRT5 plays vital roles in the regulation of autoimmune responses, cell division, metabolism, genome stability and cellular senescence [48, 49], but little research has been done on whether and how SIRT5 functions in ischaemic stroke. Diaz-Canestro et al. found that SIRT5 could promote cerebral I/R injury-induced BBB damage [27]. However, other studies showed that SIRT5-mediated desuccinylation of lysine protected against mitochondrial metabolic disorder in mice after subarachnoid haemorrhage [28]. This study demonstrated that SIRT5 mediated ANXA1 desuccinylation, leading to its nuclear translocation and activating the microglial-induced inflammatory response. In addition, in vivo-specific microglial SIRT5 knockdown showed neuroprotective and cognitive-preserving effects against ischaemic brain injury, as proven by ischaemic volume reduction, neurological function score decrease, and cognitive function improvement. Nevertheless, as SIRT5 is not expressed only in microglia, we cannot exclude the possibility that the impact of SIRT5 on cerebral ischaemia stems from the possible synergistic action of multiple cells in ischaemic tissue, such as neurons, astrocytes and oligodendrocytes. Therefore, the effects of SIRT5 on these cells remain to be further investigated in future studies.

Previous studies have shown that ANXA1 exerts different biological roles depending on its subcellular localization. ANXA1 membrane trafficking and release exert anti-inflammatory effects and protect neurons against OGD/R-induced injury [13]. However, there is evidence suggesting that nuclear translocation of ANXA1 can enhance the production of proinflammatory factors in microglia following ischaemic stroke [15]. In this study, we demonstrate that the succinylation state of ANXA1 regulates its subcellular location. Indeed, we revealed that desuccinylation of ANXA1 promotes its nuclear localization and decreases its membrane trafficking and release. Furthermore, SIRT5-mediated desuccinylation of ANXA1 blocks its interaction with FPR2. Reversible PTMs strictly regulate protein function, which creates an on or off state important for many biological processes. Many proteins are dynamically modified at multiple sites through different modifications. The crosstalk between different modifications, which affects the function of proteins synergistically or antagonistically, has been studied extensively [50]. A recent study reported that SENP6-dependent deSUMOylation of ANXA1 is crucial for its nuclear translocation [18]. In this study, we found that SIRT5-mediated desuccinylation of ANXA1 could promote the interaction of SENP6 with ANXA1 and decrease the SUMOylation level of ANXA1. TRPM7-, EGFR- and PKC-dependent phosphorylation has also been verified to have critical effects on the regulation of ANXA1 subcellular localization [51, 52]. Based on the current study, ANXA1 can be modified by succinylation; thus, it will be interesting to further investigate how phosphorylation, SUMOylation and succinylation comodulate the subcellular localization of ANXA1.

Microglia, as the main immune cells of the CNS, play a critical role in the regulation of neuroinflammation associated with the occurrence and development of ischaemic stroke. Hyperactivation of microglia leads to overexpression and secretion of proinflammatory cytokines and chemokines [53, 54]. Our research demonstrated that SIRT5 knockdown could prevent microglial overactivation and lessen the expression of proinflammatory factors, such as TNF-α, IL-6, IL-1β, CCL2 and CXCL1. In the present study, to clarify the function of SIRT5 in microglia, we did both the in vivo studies in mice underwent MCAO/R surgery and in vitro studies in primary cultured microglia. As we could not overlook the notion of microglial heterogeneity and microglial states are modulated by local cues [55, 56], even though we obtained similar results from the in vivo and in vitro studies, we should certainly notice the heterogeneity of primary cultured microglia and in vivo microglia. On the other hand, large amounts of empirical evidence now available show that microglial phagocytosis plays a key role in the recovery of neurological function during cerebral damage [57]. Ischaemic stroke results in massive brain cell death, and microglia can phagocytize and remove dead cells and debris. Whether SIRT5 is also involved in regulating microglial phagocytosis after ischaemic stroke remains unclear. Our research only explored the impact of SIRT5 on the expression and release of proinflammatory cytokines; the effect of SIRT5 on microglial phagocytosis after ischaemic stroke needs further research.

In this study, infarct models of mice with thread embolism were applied to simulate an acute ischaemic stroke model. Our research revealed that microglia-specific SIRT5 knockdown decreased the cerebral infarction volume and the nerve dysfunction scores. Behavioural experiments also suggested that SIRT5 knockdown obviously ameliorated the learning and memorizing abilities of mice with ischaemic stroke. Since AAV-mediated gene expression in vivo takes some time, the mice were pretreated with AAV injection for 4 weeks before MCAO surgery. Therefore, more detailed work is needed to explore the therapeutic effect posttreatment. On the other hand, Cx3cr1-Cre mice express Cre recombinase under the Cx3cr1 promoter, not only in microglia but also in other mononuclear phagocyte systems, including the monocyte and macrophage compartments [58]. Consequently, AAV particles may also infect macrophages and infiltrate monocytes, and the effects of AAV particle injections on infiltrating macrophages and monocytes remain to be further investigated.

## Conclusions

**Fig. 10:**
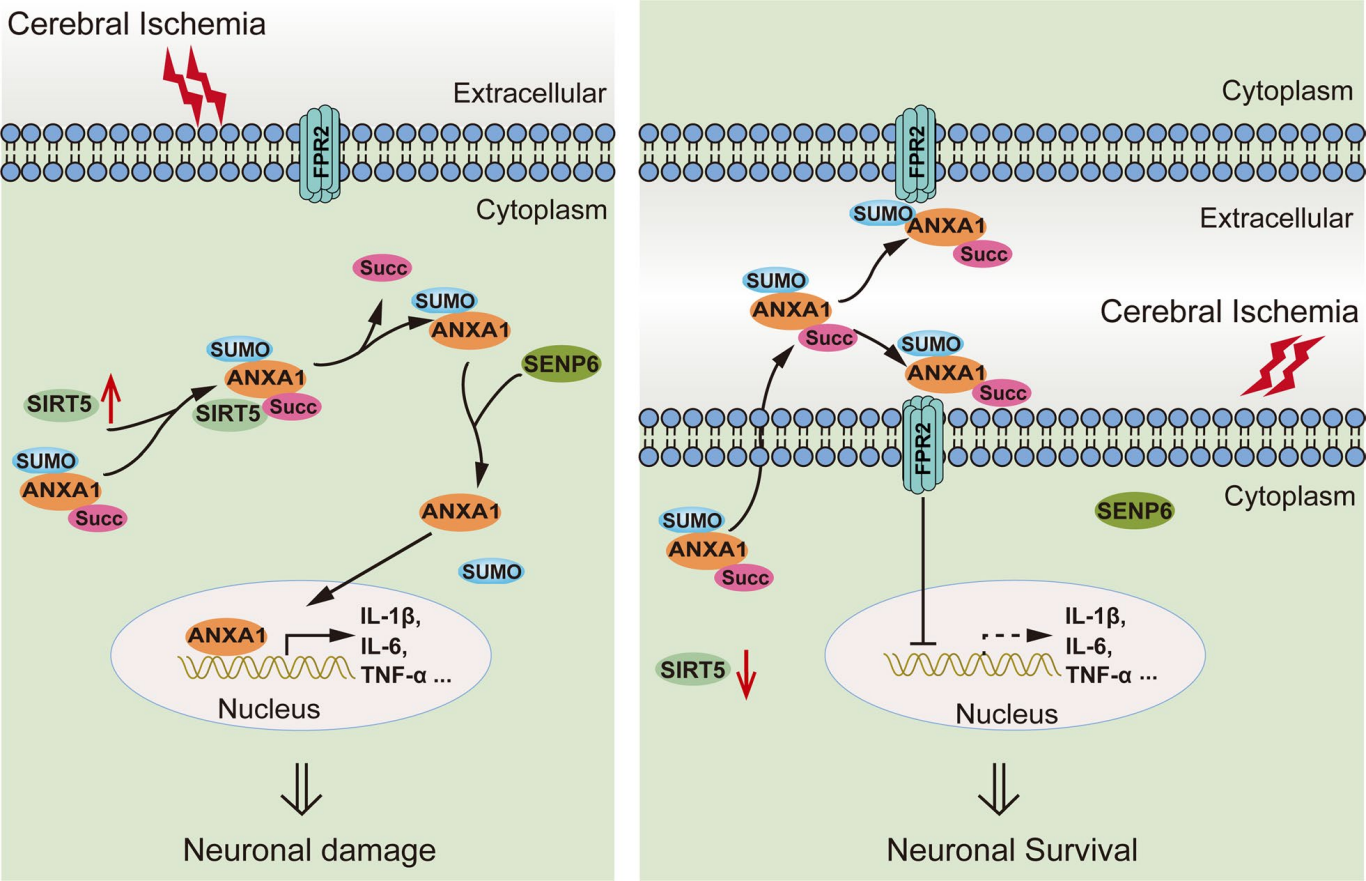
Schematic diagram representing the mechanism by which SIRT5 inhibition protects neurons against cerebral I/R injury by regulating microglia-induced neuroinflammation. The increase in SIRT5 in microglia induced by ischaemic stroke causes ANXA1 desuccinylation, which decreases ANXA1 membrane recruitment and secretion but promotes ANXA1 nuclear translocation, resulting in the production of proinflammatory mediators and ultimately enhancing neuroinflammation damage. Thus, SIRT5 ablation might protect against neuronal damage induced by cerebral I/R injury

In summary, this study elucidates a previously unrecognized effect and molecular mechanism by which SIRT5 is involved in neuroinflammation induced by microglia following cerebral I/R injury. We have provided convincing evidence that SIRT5 acts as a desuccinylase to mediate ANXA1 desuccinylation, thereby promoting ANXA1 translocation to the nucleus and decreasing its membrane transport and extracellular secretion, resulting in the hyperactivation of microglia and the overrelease of proinflammatory cytokines and chemokines, ultimately leading to neuronal cell damage after ischaemic stroke (Fig. 10). In conclusion, this research investigates a previously unrecognized role of SIRT5 and reveals that targeting SIRT5 and/or its interaction with ANXA1 in microglia might be a promising potential therapeutic strategy for ischaemic stroke and probably other kinds of neurological diseases with neuroinflammatory manifestations.

## Supplementary Information

**Additional file 1: Figure S1.** SIRT5 inhibition upregulates the succinylation level of ANXA1 in microglia subjected to OGD/R. Primary cultured microglia were treated with vehicle or MC3482 and then subjected to OGD/R for 24 h. Co-IP analysis showing the succinylation level of ANXA1. Data represent three independent experiments.

**Additional file 2: Figure S2.** Schematic of human ANXA1 protein and amino acid sequence alignment of ANXA1 sequences from different species as indicated. The conserved succinylation motif lysins are shown.

**Additional file 3: Figure S3.** (A) qPCR assay indicating *Sirt5* mRNA expression in the contralateral and ipsilateral hemisphere tissues at 24 h after reperfusion. *n* = 3. (B, C) Mouse brain homogenates were extracted in the contralateral and ipsilateral hemisphere tissues at 24 h of reperfusion after 1 h of MCAO surgery. Western blot assay (B) and quantification analysis (C) showing SIRT5 protein expression; *n* = 3. Data are presented as the mean ± SEM. ***P < 0.001, and ****P < 0.0001.

**Additional file 4: Figure S4.** (A) Immunofluorescence analysis shows the purity of primary cultured microglial cells. Cells were fixed and stained for microglia-specific marker Iba1 (red) with colabelled 4′,6-diamidino-2-phenylindole (DAPI; blue, nuclei). Scale bar, 20 μm. (B) Primary cultured microglia were subjected to OGD and reoxygenation at 24 h. Immunofluorescence assays of SIRT5 (green) and Iba1 (red) with DAPI (blue, nuclei). Scale bars, 40 μm. Data represent three independent experiments.

**Additional file 5: Figure S5.** Representative immunoblotting of SIRT5 (A) and quantification of SIRT5 expression (B) in primary cultured microglial cells transduced with recombinant adenovirus carrying the SIRT5 coding sequence or shRNA sequence for 48 h (*n* = 3). All data are presented as the mean ± SEM. ***P < 0.001.

**Additional file 6: Figure S6.** Representative immunoblotting of SIRT5 (A) and quantification of SIRT5 expression (B) in the isolated microglial cells from Cx3cr1-Cre mice injected with AAV at 4 weeks (*n* = 3). All data are presented as the mean ± SEM. **P < 0.01 and ****P < 0.0001.

**Additional file 7: Tables.** Antibodies and primers employed in this study.

**Additional file 8:** Supplementary Materials and Methods

**Additional file 9:** Full gels of the western blot images.

## Data Availability

The datasets used and/or analysed during the present study are available from the corresponding author upon reasonable request.

## Abbreviations

ANXA1: Annexin A1
BBB: Blood–brain barrier
BSA: Bovine serum albumin
Ccl2: C–C motif chemokine ligand 2
CNS: Central nervous system
Co-IP: Coimmunoprecipitation
Cxcl1: C-X-C motif chemokine ligand 1
DMEM: Dulbecco’s modified Eagle’s medium
FBS: Foetal bovine serum
HA: Haemagglutinin
Iba1: Ionized calcium binding adaptor molecule-1
IL-1β: Interleukin 1-beta
IL-6: Interleukin 6
iNOS: Inducible nitric oxide synthase
I/R: Ischaemia–reperfusion
MCAO: Middle cerebral artery occlusion
mNSS: Modified neurological severity score
MWM: Morris water maze
OGD/R: Oxygen–glucose deprivation and reoxygenation
PTMs: Posttranslational modifications
SIRT5: Sirtuin 5
TNF-α: Tumour necrosis factor alpha
