# Supplementary figures and images for: Sirtuin 5 aggravates microglia-induced neuroinflammation following ischaemic stroke by modulating the desuccinylation of Annexin-A1
Source: J Neuroinflammation. 2022 Dec 14;19:301. doi: 10.1186/s12974-022-02665-x (PMC9753274; doi:10.1186/s12974-022-02665-x)

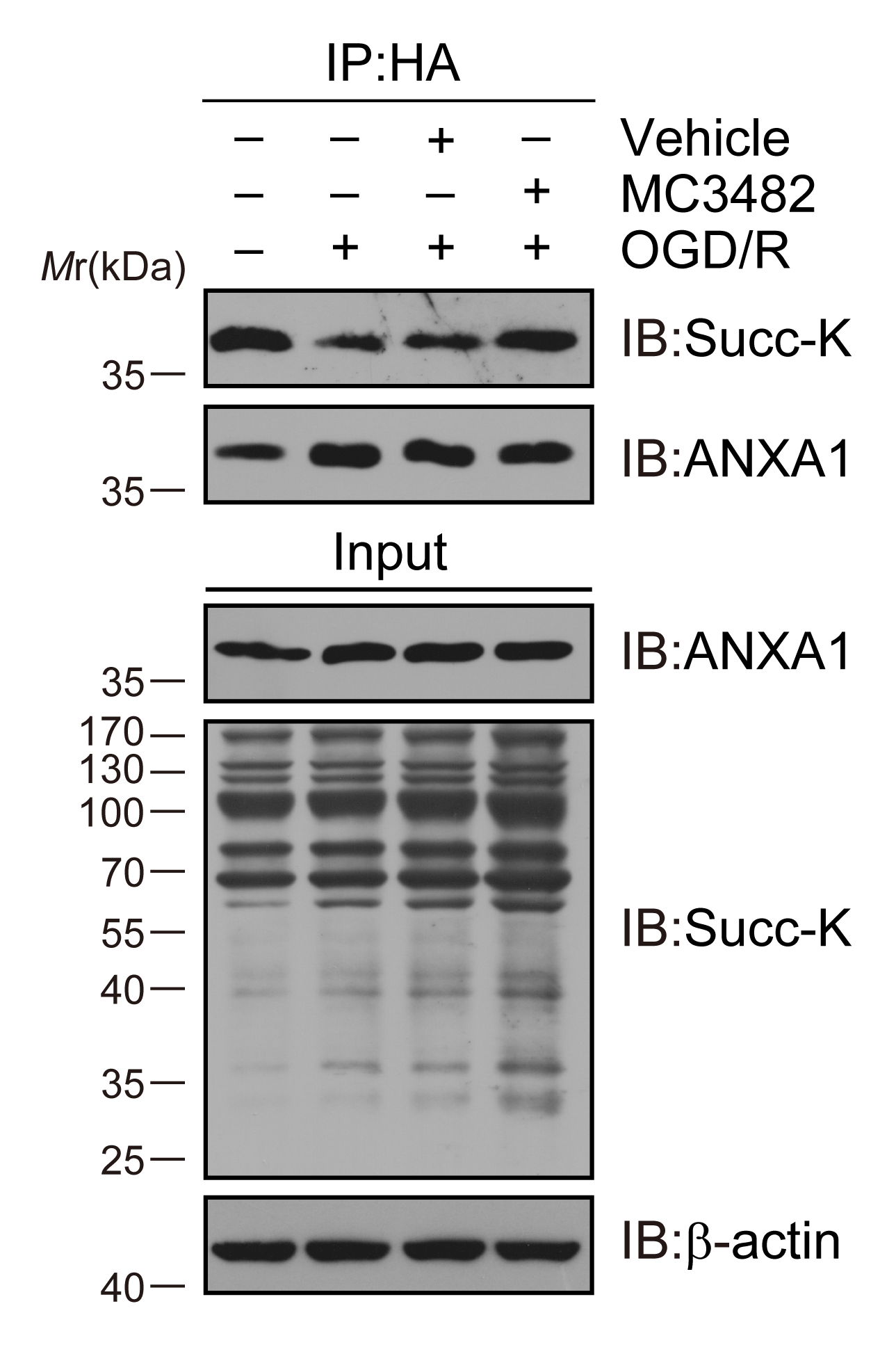

Supplement: Supplementary file 1 — Additional file 1: Figure S1. SIRT5 inhibition upregulates the succinylation level of ANXA1 in microglia subjected to OGD/R. Primary cultured microglia were treated with vehicle or MC3482 and then subjected to OGD/R for 24 h. Co-IP analysis showing the succinylation level of ANXA1. Data represent three independent experiments. [file 12974_2022_2665_MOESM1_ESM.tif]

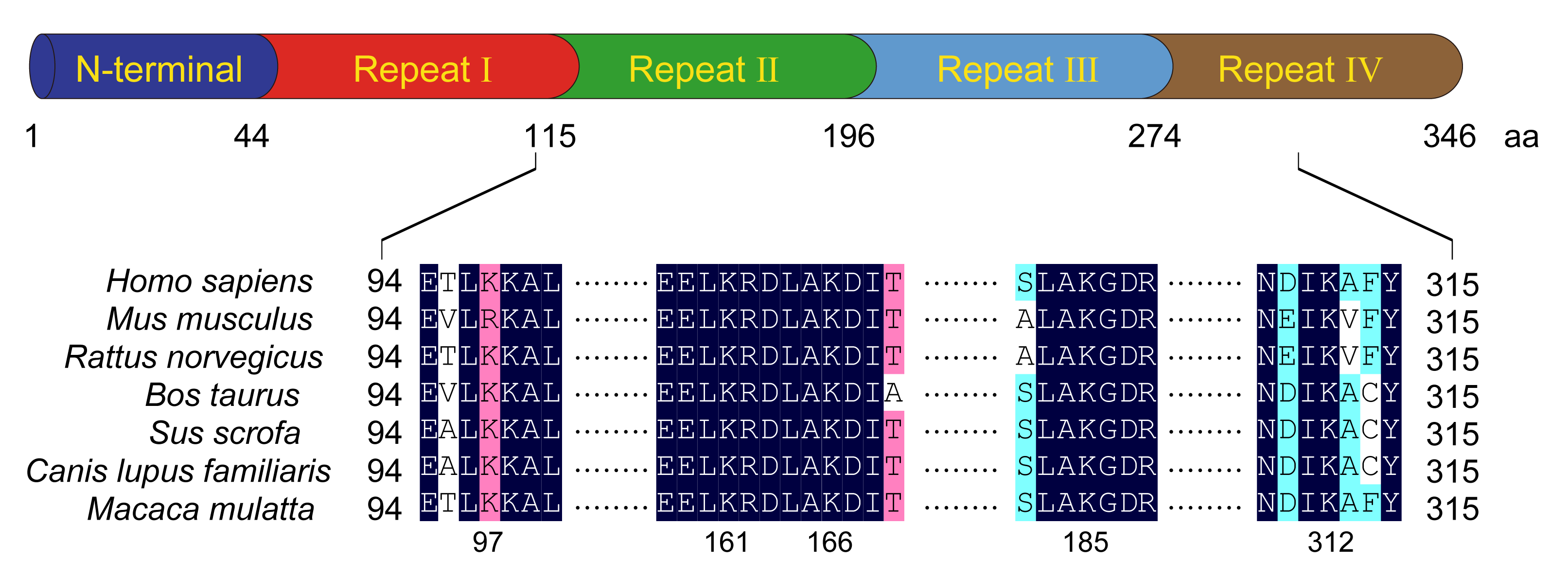

Supplement: Supplementary file 2 — Additional file 2: Figure S2. Schematic of human ANXA1 protein and amino acid sequence alignment of ANXA1 sequences from different species as indicated. The conserved succinylation motif lysins are shown. [file 12974_2022_2665_MOESM2_ESM.tif]

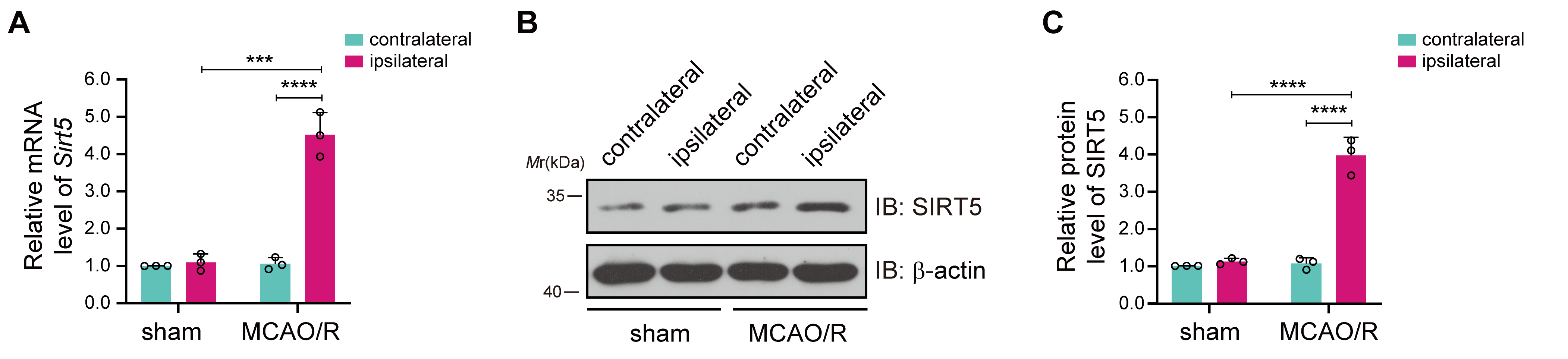

Supplement: Supplementary file 3 — Additional file 3: Figure S3. (A) qPCR assay indicating Sirt5 mRNA expression in the contralateral and ipsilateral hemisphere tissues at 24 h after reperfusion. n = 3. (B, C) Mouse brain homogenates were extracted in the contralateral and ipsilateral hemisphere tissues at 24 h of reperfusion after 1 h of MCAO surgery. Western blot assay (B) and quantification analysis (C) showing SIRT5 protein expression; n = 3. Data are presented as the mean ± SEM. ***P < 0.001, and ****P < 0.0001. [file 12974_2022_2665_MOESM3_ESM.tif]

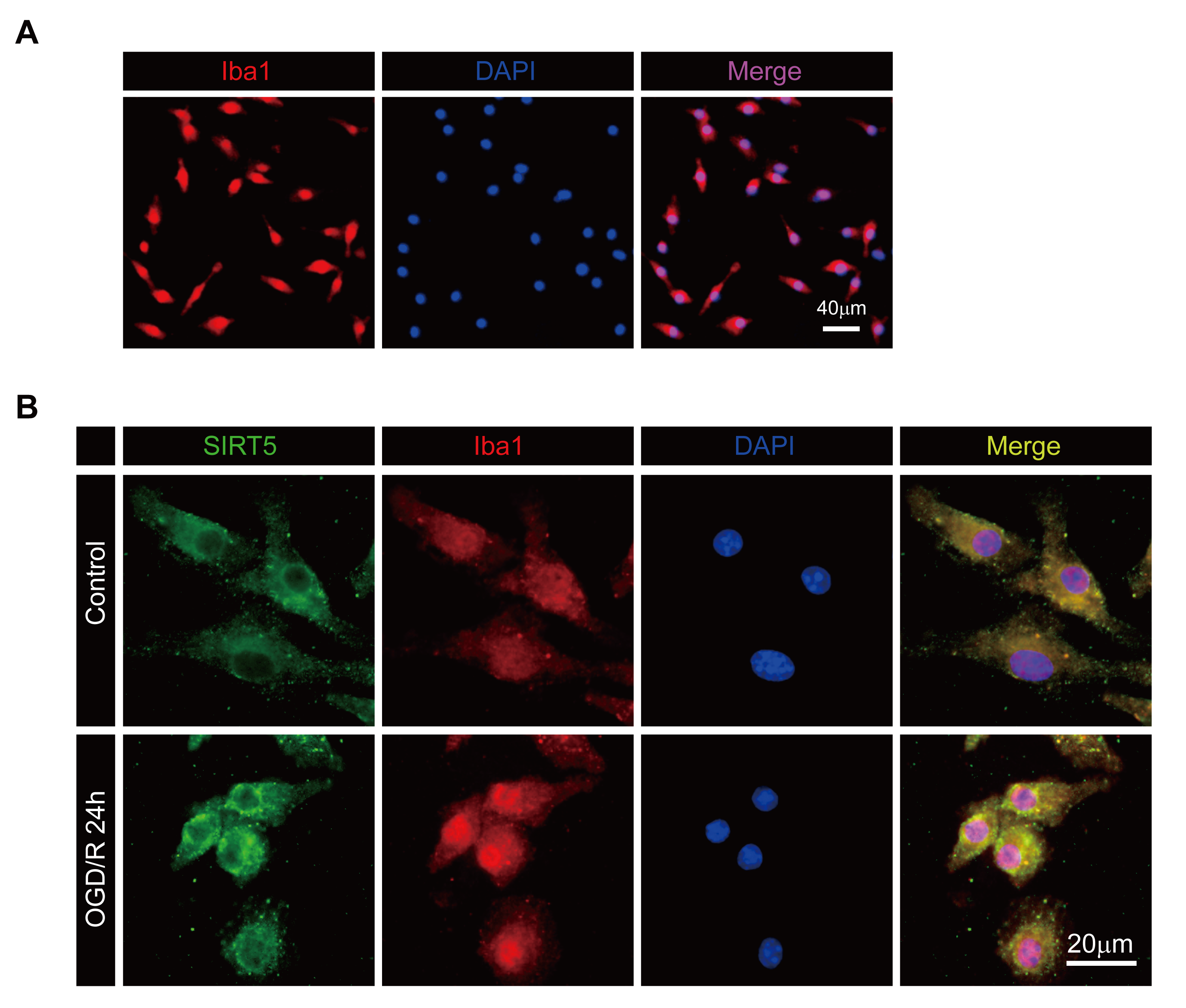

Supplement: Supplementary file 4 — Additional file 4: Figure S4. (A) Immunofluorescence analysis shows the purity of primary cultured microglial cells. Cells were fixed and stained for microglia-specific marker Iba1 (red) with colabelled 4′,6-diamidino-2-phenylindole (DAPI; blue, nuclei). Scale bar, 20 μm. (B) Primary cultured microglia were subjected to OGD and reoxygenation at 24 h. Immunofluorescence assays of SIRT5 (green) and Iba1 (red) with DAPI (blue, nuclei). Scale bars, 40 μm. Data represent three independent experiments. [file 12974_2022_2665_MOESM4_ESM.tif]

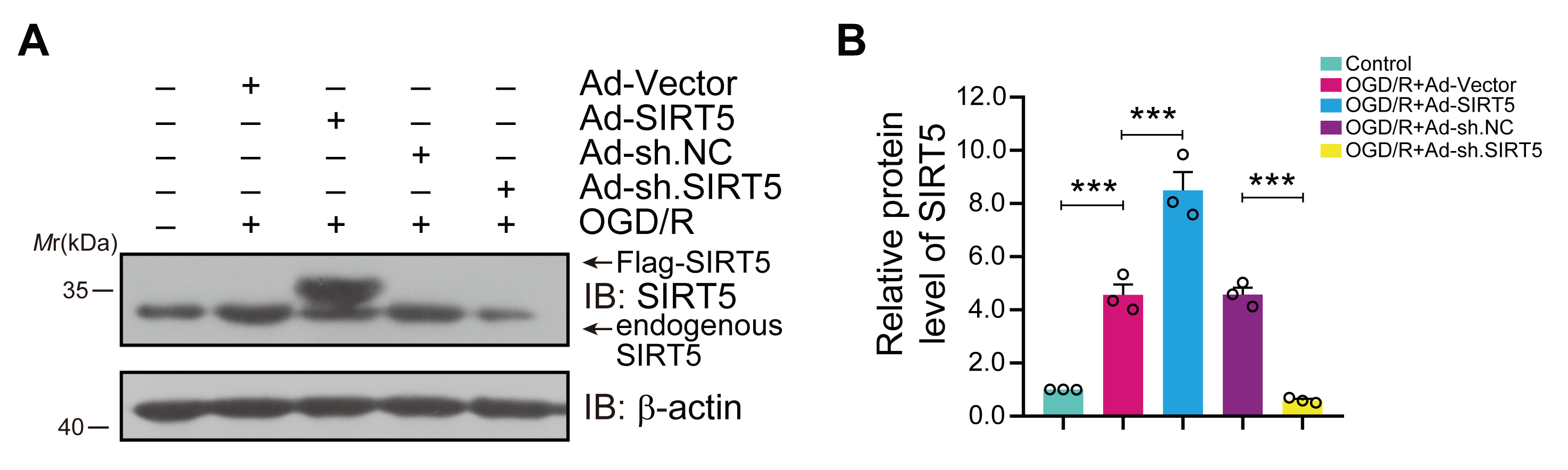

Supplement: Supplementary file 5 — Additional file 5: Figure S5. Representative immunoblotting of SIRT5 (A) and quantification of SIRT5 expression (B) in primary cultured microglial cells transduced with recombinant adenovirus carrying the SIRT5 coding sequence or shRNA sequence for 48 h (n = 3). All data are presented as the mean ± SEM. ***P < 0.001. [file 12974_2022_2665_MOESM5_ESM.tif]

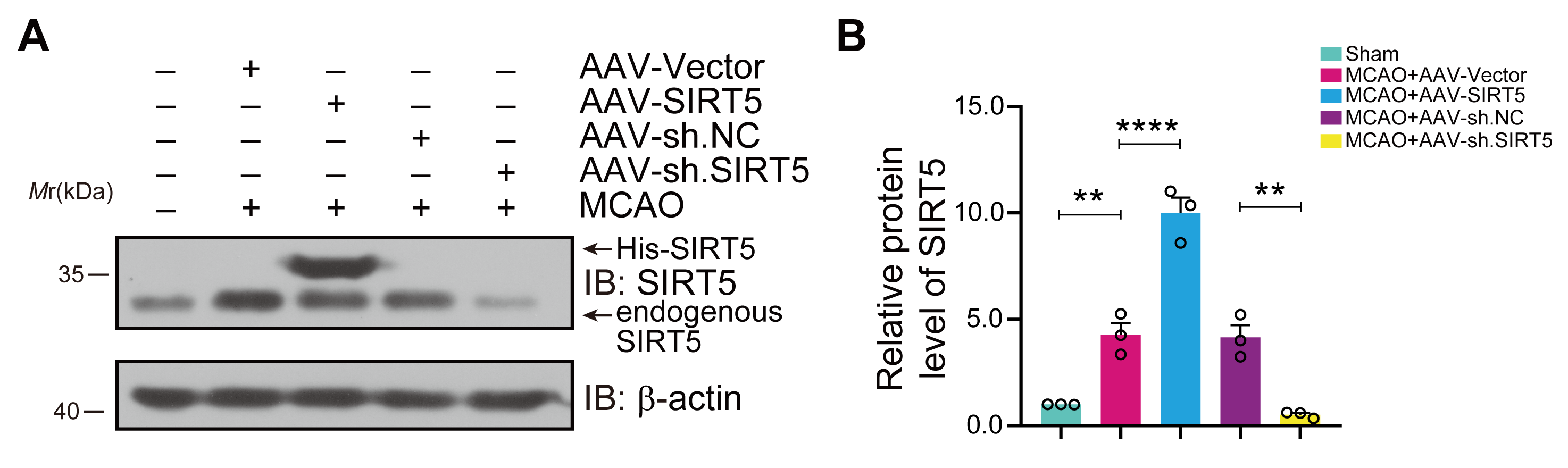

Supplement: Supplementary file 6 — Additional file 6: Figure S6. Representative immunoblotting of SIRT5 (A) and quantification of SIRT5 expression (B) in the isolated microglial cells from Cx3cr1-Cre mice injected with AAV at 4 weeks (n = 3). All data are presented as the mean ± SEM. **P < 0.01 and ****P < 0.0001. [file 12974_2022_2665_MOESM6_ESM.tif]
