# Supplementary material for: Sirtuin 5 aggravates microglia-induced neuroinflammation following ischaemic stroke by modulating the desuccinylation of Annexin-A1
Source: J Neuroinflammation. 2022 Dec 14;19:301. doi: 10.1186/s12974-022-02665-x (PMC9753274; doi:10.1186/s12974-022-02665-x)
Supplement: Supplementary file 7 — Additional file 7: Tables. Antibodies and primers employed in this study. [file 12974_2022_2665_MOESM7_ESM.docx]

**Table S1. Antibodies employed in this study.**

| Antibody | Species | Type | IB | IF | Source | Identifier |
| --- | --- | --- | --- | --- | --- | --- |
| SIRT5 | Rabbit | Poly- | 1:1000 | 1:50 | Proteintech | 15122-1-AP |
| ANXA1 | Mouse | Mono- | 1:1000 | 1:50 | Santa Cruz | sc-12740 |
| Succ-K | Rabbit | Poly- | 1:1000 |  | PTMBIO | PTM-401 |
| HA | Rabbit | Poly- | 1:1000 | 1:200 | Proteintech | 51064-2-AP |
| Flag | Mouse | Mono- | 1:2000 |  | Santa Cruz | sc-166355 |
| β-actin | Mouse | Mono- | 1:1000 |  | Santa Cruz | sc-47778 |
| Na^+^-K^+^/ATPase | Rabbit | Poly- | 1:1000 |  | Cell signaling | #9339 |
| Histone H3 | Rabbit | Mono- | 1:2000 |  | Cell Signaling | #4499 |
| FPR2 | Mouse | Mono- | 1:1000 |  | Santa Cruz | sc-100585 |
| SUMO-2/3 | Rabbit | Mono- | 1:1000 |  | Cell signaling | #4971 |
| SENP6 | Mouse | Mono- | 1:1000 |  | Santa Cruz | sc-100585 |
| iNOS | Mouse | Mono- | 1:1000 | 1:100 | Santa Cruz | sc-7271 |
| Iba1 | Rabbit | Poly- |  | 1:500 | Wako | #019-19741 |
| CD16/32 | Mouse | Poly- | 1:500 |  | R&D systems | AF1460 |
| cleaved caspase-3 | Rabbit | Mono- | 1:1000 |  | Cell Signaling | #9664 |
| cleaved caspase-9 | Rabbit | Mono- | 1:1000 |  | Cell Signaling | #20750 |
| cleaved PARP | Rabbit | Mono- | 1:1000 |  | Cell Signaling | #5625 |
| Anti-rabbit IgG | Goat | Poly- | 1:10000 |  | Cell Signaling | #7074 |
| Anti-mouse IgG | Horse | Poly- | 1:10000 |  | Cell Signaling | #7076 |

Abbreviations: IB, Immunoblotting; IF, Immunofluorescence.

**Table S2. Primers used in this study.**

| Primer name | Primer sequences (5’- 3’) | |
| --- | --- | --- |
|  | Forward | Reverse |
| Quantitative RT-PCR primers | | |
| *Sirt5* | GCCACCGACAGATTCAGGTT | CCACAGGGCGGTTAAGAAGT |
| *Il-1β* | GAAAGACGGCACACCCAC | TGTGACCCTGAGCGACCT |
| *Il-6* | TCTCTGGGAAATCGTGGAA | GATGGTCTTGGTCCTTAGCC |
| *Tnf-α* | ACGGCATGGATCTCAAAGAC | AGATAGCAAATCGGCTGACG |
| *Cxcl1* | GAGCTTGAAGGTGTTGCCCT | CGCGACCATTCTTGAGTGTG |
| *Ccl2* | GCAGGTCCCTGTCATGCTTC | GTGGGGCGTTAACTGCATCT |
| *β-actin* | TTCGTTGCCGGTCCACACCC | GCTTTGCACATGCCGGAGCC |
| Genotyping primers | | |
| *Cx3cr1* Cre | CAACGAGTGATGAGGTTCGCAAG | ACACCAGAGACGGAAATCCATCG |
