# Supplementary material for: Sirtuin 5 aggravates microglia-induced neuroinflammation following ischaemic stroke by modulating the desuccinylation of Annexin-A1
Source: J Neuroinflammation. 2022 Dec 14;19:301. doi: 10.1186/s12974-022-02665-x (PMC9753274; doi:10.1186/s12974-022-02665-x)
Supplement: Supplementary file 8 — Additional file 8: Supplementary Materials and Methods [file 12974_2022_2665_MOESM8_ESM.docx]

**Additional Materials and Methods**

**Co-immunoprecipitation**

Immunoassay of the succinylation of ANXA1 was analyzed by immunoprecipitation with antibody to ANXA1 or HA followed by immunoblot analysis with antibody to Succ-K and to ANXA1 (loading control) as previously described. Briefly, Fresh samples were collected and proteins concentration were detected using BCA protein concentration determination kit (Beyotime Biotechnology, Shanghai, China). The supernatants were incubated with anti-ANXA1 or HA at 4 °C overnight. Protein A/G Plus-agarose beads (Beyotime) were added to the samples and incubated for 2 h at room temperature. Then washed times with 0.01 mol/L PBS, mixed with 2 × loading buffer, boiled, and run on SDS-PAGE, followed by transfering to PVDF membranes for immunoblotting. Aliquots of the original lysates were also run on SDS-PAGE in parallel for immunoblotting to determine the amount of input. The band intensities of western blots were analyzed by ImageJ Software (NIH, Baltimore, MD, USA) with background subtraction.

**Plasmid constructs and transfection**

Plasmids were constructed as we previously described. Briefly, the full-length DNA segment ANXA1 coding sequence was amplified by PCR, and cloned into HA-tagged pcDNA 3.0 (HA-ANXA1). For Flag-SIRT5 and Flag-SIRT7 plasmid, PCR was applied to amplify the full-length cDNA of human SIRT5 and SIRT7, and cloned into indicated vectors including pFlag-CMV2, respectively. For site-directed mutagenesis, the subsequent mutants ANXA1-K97R, K161R, K166R, K195R, K312R (lysine-to-arginine), K166E (lysine-to-glutamicacid) and SIRT5-H158Y (histidine-to-tyrosine) were constructed using homologous recombination via Trelief SoSoo Cloning Kit Ver.2 (TSINGKE, Beijing, China) reference to product specification. All constructs were confirmed by DNA sequencing analysis (performed by Sangon Biotechnology, Shanghai, China). Human SIRT5 shRNA plasmids for HEK293T cell transfection were purchased from Genepharma (Shanghai, China). The target sequence for SIRT5 (GenBank NM_001193267.2) shRNA no. 1 was 5′- GCCCTTGAACATTTCCCAATG -3′, no. 2 was 5′- GCATTAGAACTACAGACAAC-3′, and a Scr shRNA served as a scramble control group. The recombinant plasmids were transfected into cells using Lipofectamine 3000 (Invitrogen, NY, USA) when the cells were 80 to 90% confluent, following the manufacturer’s instructions.

**Enzyme-linked immunosorbent assay (ELISA)**

The expression of proinflammatory cytokines and chemokines IL-1β, IL-6, TNF-α, CXCL1, CCL2 in the supernatant of treated and untreated microglia cells cultures was determined with a mouse enzyme-linked immunosorbent assay (ELISA) kit (R&D Systems, Wiesbaden, Germany), and ANXA1 release was measured using an ELISA kit purchased from Cloud-Clone Corp (SEE787Mu, Wuhan, China) according to manufacturer procedures, and results were raised as picogram or nanogram per milliliter, respectively. Both standards and samples were run in triplicate. Samples were collected in pyrogen/endotoxin-free tubes. The optical density at 450 nm was calculated by subtracting the background, and standard curves were plotted.

**TTC Staining**

At 24 hours after MCAO surgery, the mice were euthanized, and the brain was removed quickly and refrigerated at −80 °C for 5 minutes, then slice the mouse brain into six 2-millimeter-thick slices by a mouse brain matrix (RWD Life Science, Shenzhen, China). Next, dyed the sections by 2% TTC (Sigma-Aldrich, Shanghai, China) at 37°C for 20 min and then fixed with 4% paraformaldehyde. Last, analyzed the infarct volume through ImageJ software. The infarct size was measured and indicated as: Infarct size (%) = (contralateral area-ipsilateral non-infarct area) / contralateral area×100%.

**TUNEL staining**

Cell apoptosis was detected by TUNEL staining, and the operation was carried out according to the manufacturer's instructions (Roche, Basel, Switzerland). Washed the slides with PBS, and set with 4% PFA for 20 minutes. Incubated the probed slides with TUNEL reaction mixture in the dark, and washed with PBS, then stained the slides with DAPI for 15 minutes. Last, washed the slides with PBS 2×5 min, mount the cells with ProLong Gold Antifade reagent (Thermo Fisher Scientific, Waltham, MA, USA). Examined all images with a luminescence microscope (BX53, Olympus, Tokyo, Japan).

**Neurological Score**

A modified neurological severity score (mNSS) was used to determine the neurological dysfunction after 24 hours of surgery. The mNSS contains beam balance tests (score 0 to 6), motor tests (including flexion of forelimb, flexion of hindlimb and head movement, score 0 to 6), and reflexes absent & abnormal movements (score 0 to 2). Accumulated points of 1 to 4 indicate mild, 5 to 9 points indicates moderate, and 10 to 14 points indicates severe injury. The neurological function was evaluated by an independent researcher who is blind to the experiment.
