# Supplementary material for: Sirtuin 5 aggravates microglia-induced neuroinflammation following ischaemic stroke by modulating the desuccinylation of Annexin-A1
Source: J Neuroinflammation. 2022 Dec 14;19:301. doi: 10.1186/s12974-022-02665-x (PMC9753274; doi:10.1186/s12974-022-02665-x)

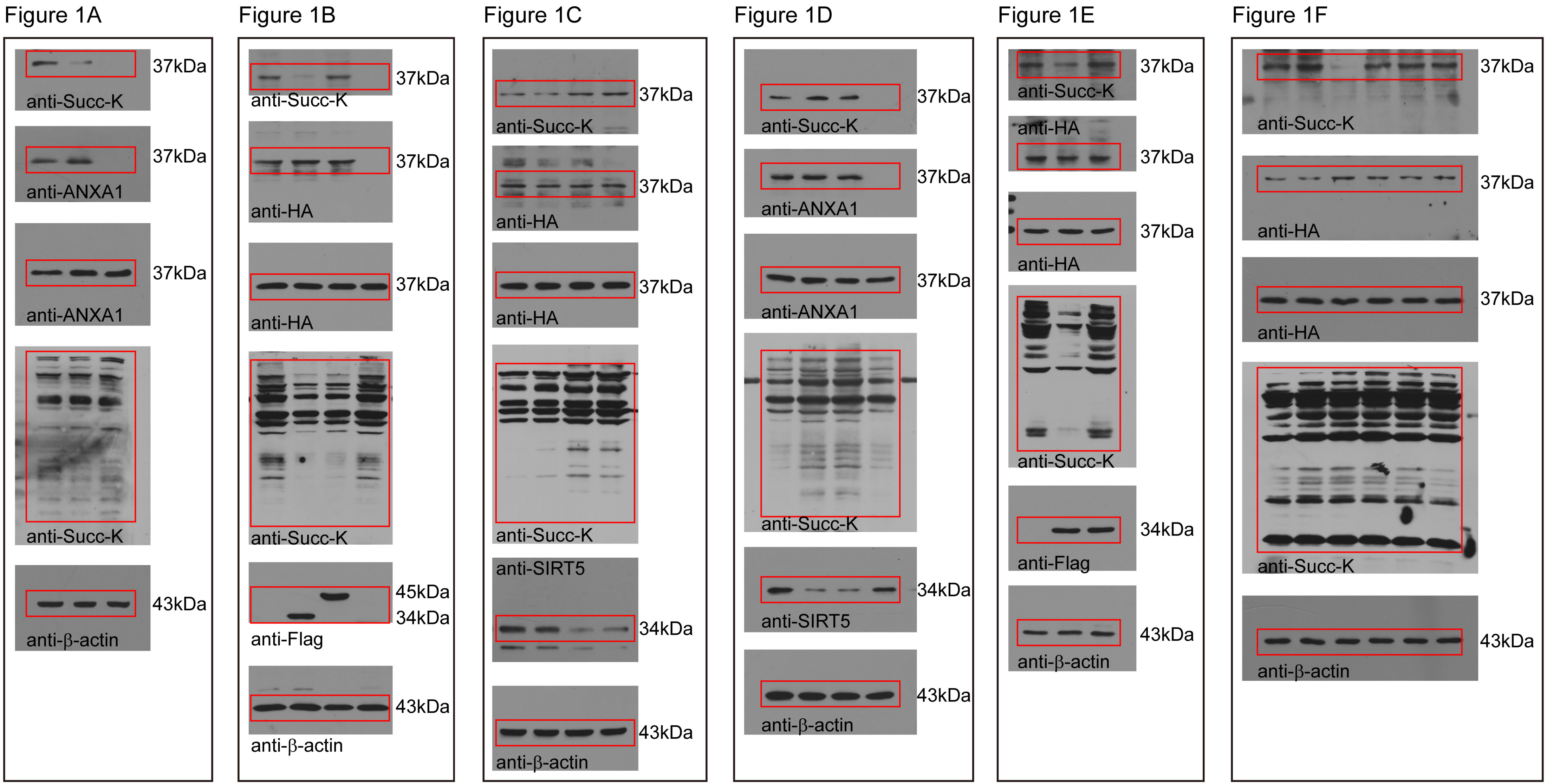

Figure 1A
anti-Succ-K
37kDa
anti-ANXA1
37kDa
anti-ANXA1
37kDa
anti-Succ-K
anti-β-actin
43kDa
Figure 1B
anti-Succ-K
37kDa
anti-HA
37kDa
anti-HA
37kDa
anti-Succ-K
anti-Flag
45kDa
34kDa
anti-β-actin
43kDa
Figure 1C
anti-Succ-K
37kDa
anti-HA
37kDa
anti-HA
37kDa
anti-Succ-K
anti-SIRT5
34kDa
anti-β-actin
43kDa
Figure 1D
anti-Succ-K
37kDa
anti-ANXA1
37kDa
anti-ANXA1
37kDa
anti-Succ-K
anti-SIRT5
34kDa
anti-β-actin
43kDa
Figure 1E
anti-Succ-K
37kDa
anti-HA
37kDa
anti-HA
37kDa
anti-Succ-K
anti-Flag
34kDa
anti-β-actin
43kDa
Figure 1F
anti-Succ-K
37kDa
anti-HA
37kDa
anti-HA
37kDa
anti-Succ-K
anti-β-actin
43kDa

Figure 2A

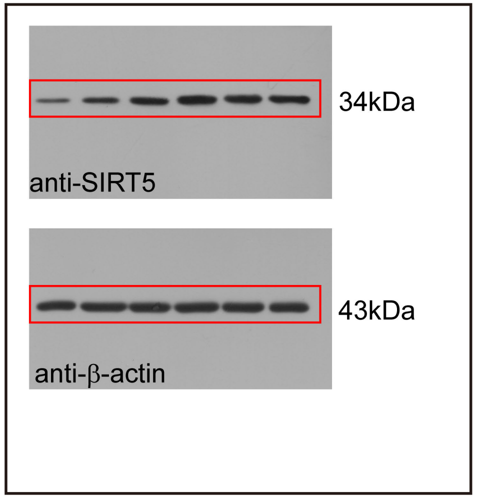

Figure 2B

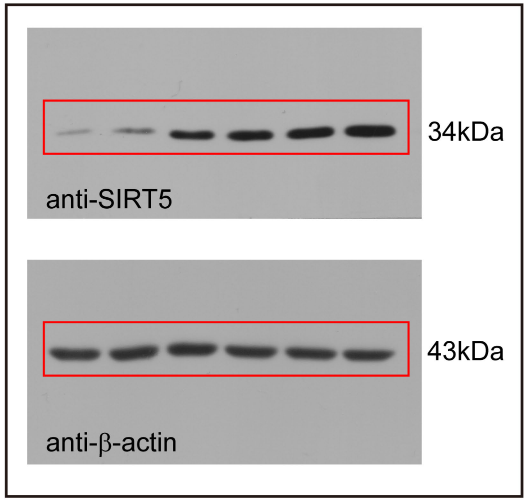

Figure 3A

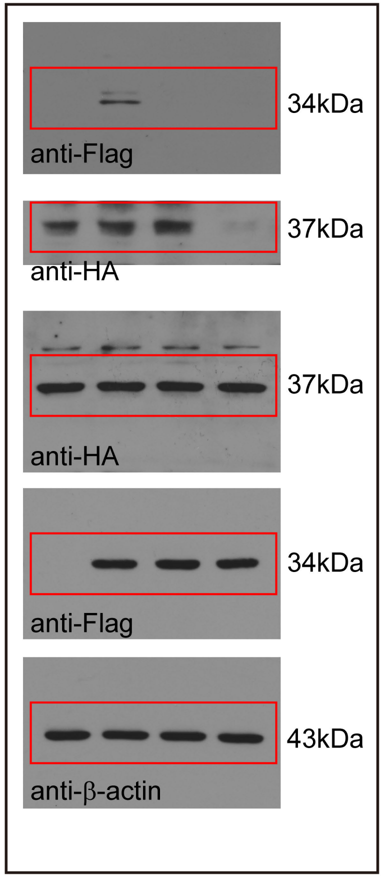

Figure 3B

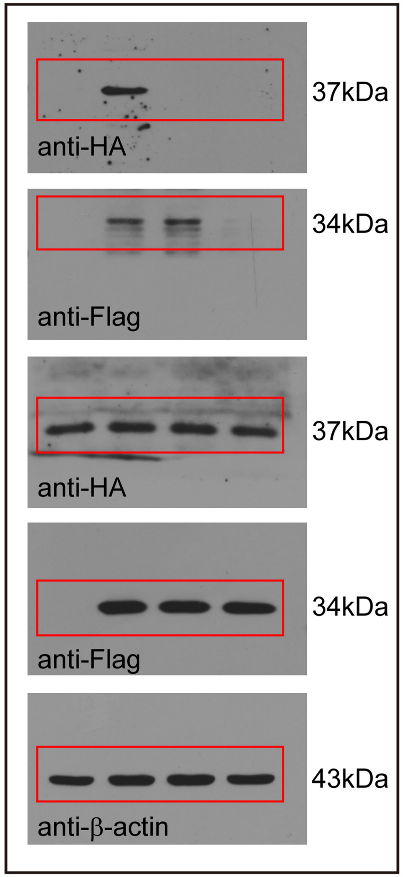

Figure 3C

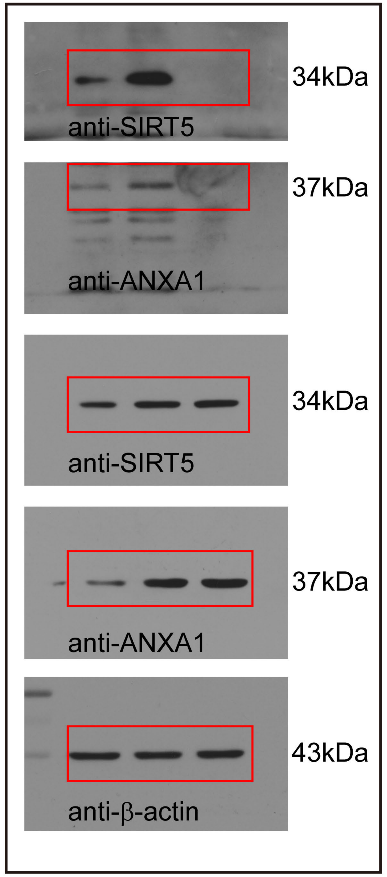

Figure 3D

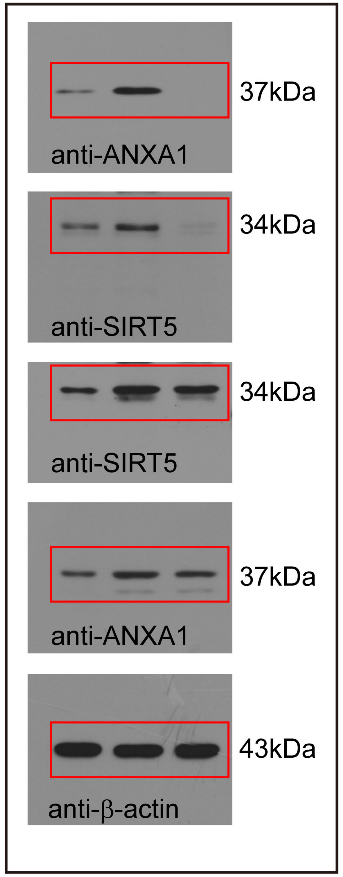

Figure 3E

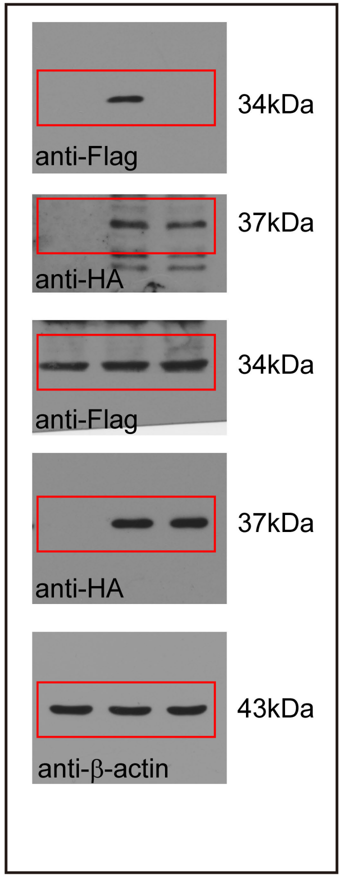

Figure 4A

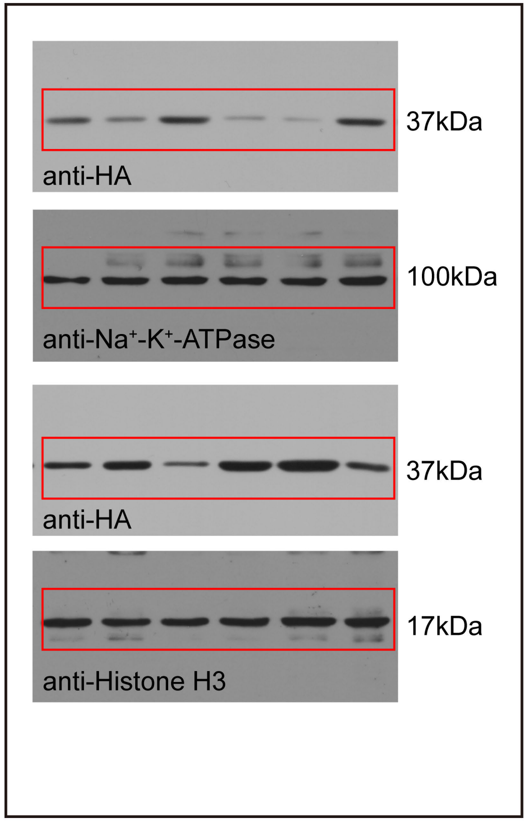

Figure 4D

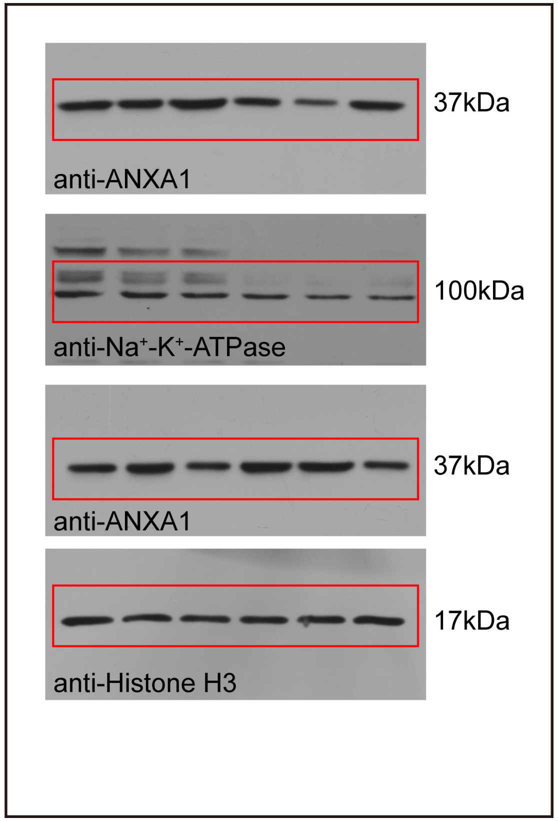

Figure 4G

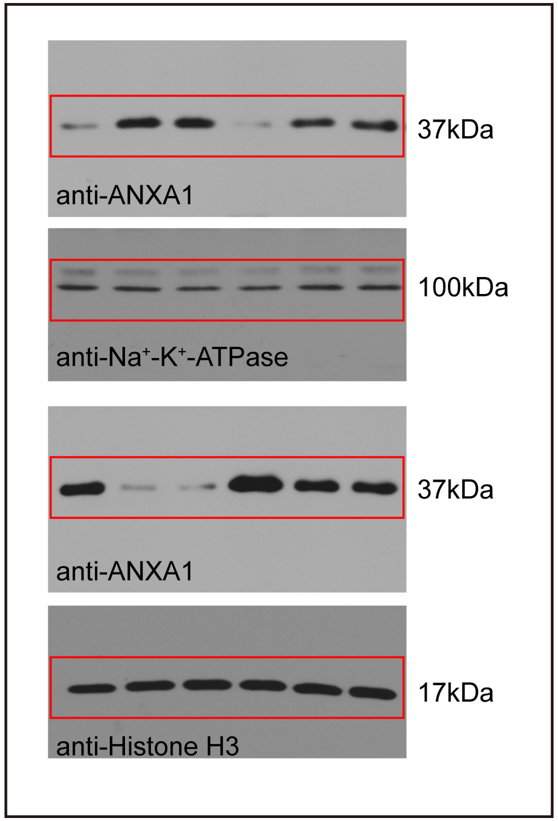

Figure 5B

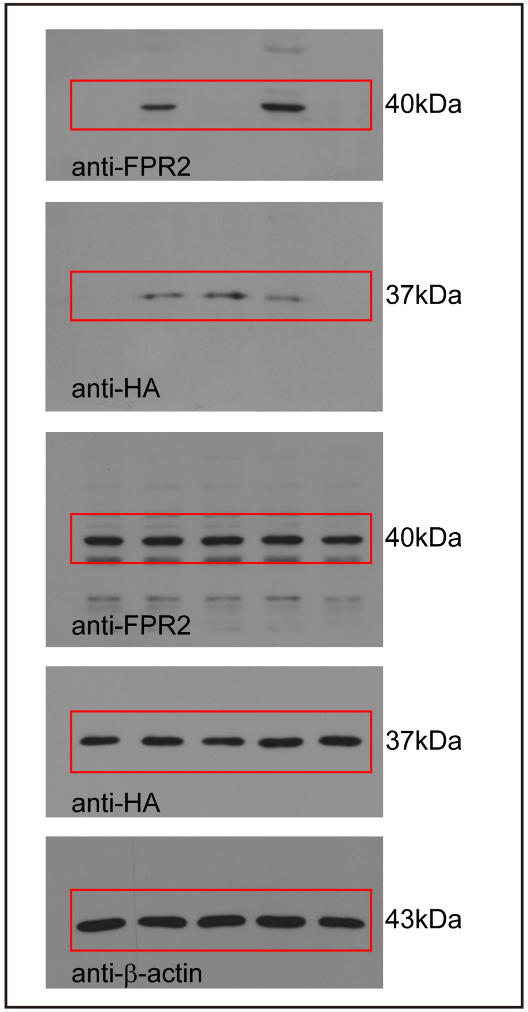

Figure 5D

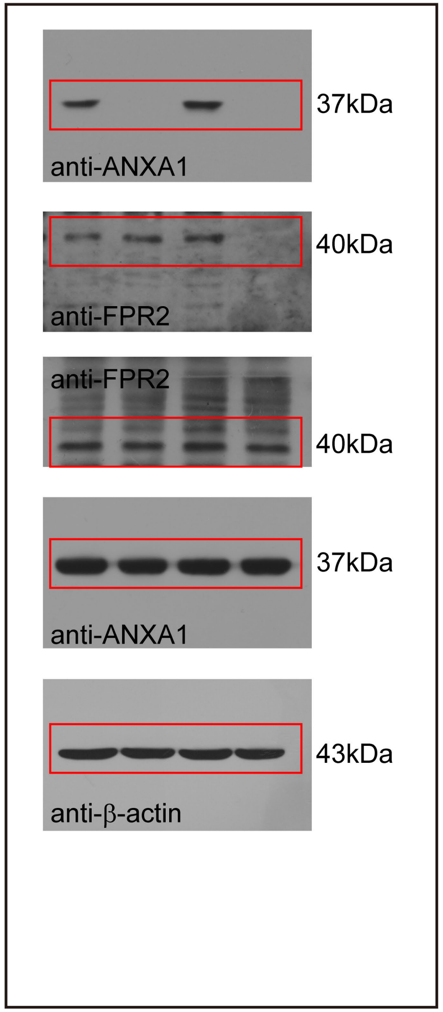

Figure 5F

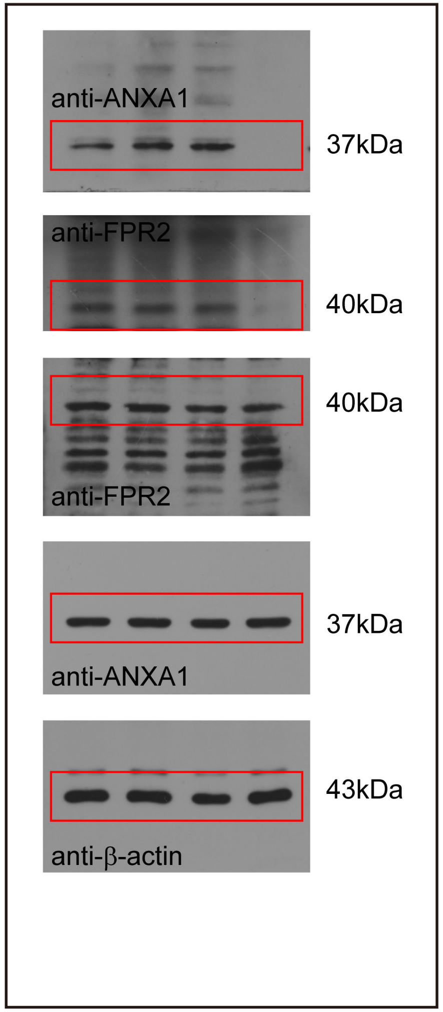

Figure 6A

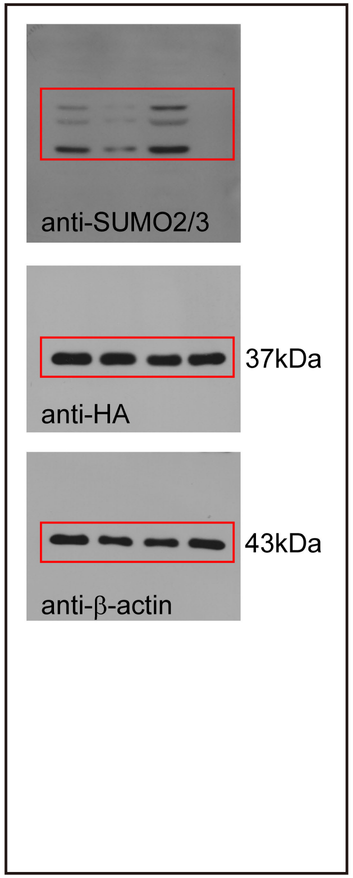

Figure 6B

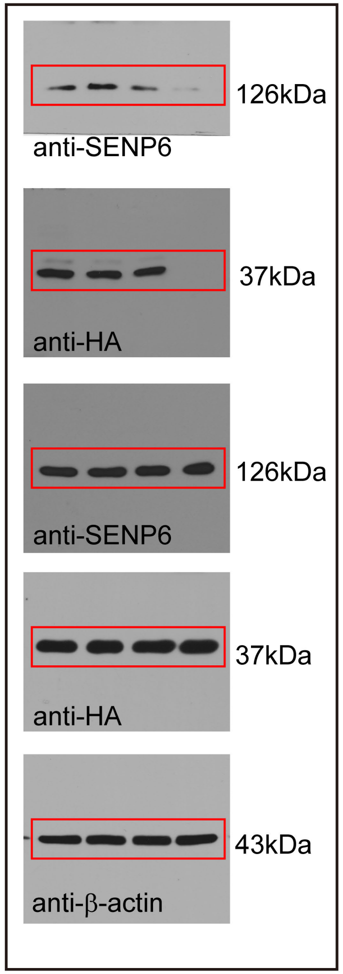

Figure 6C

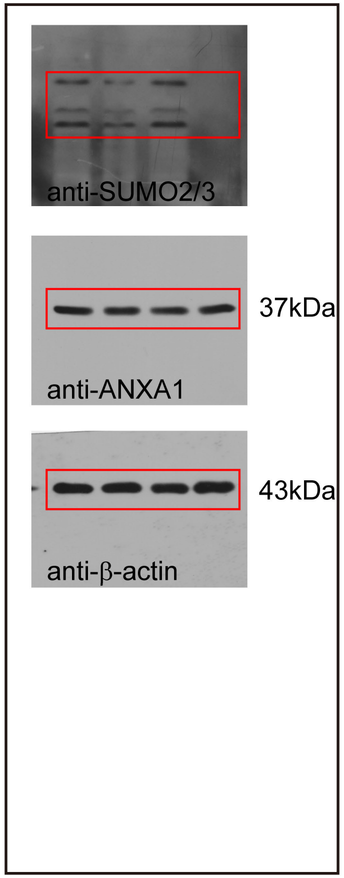

Figure 6D

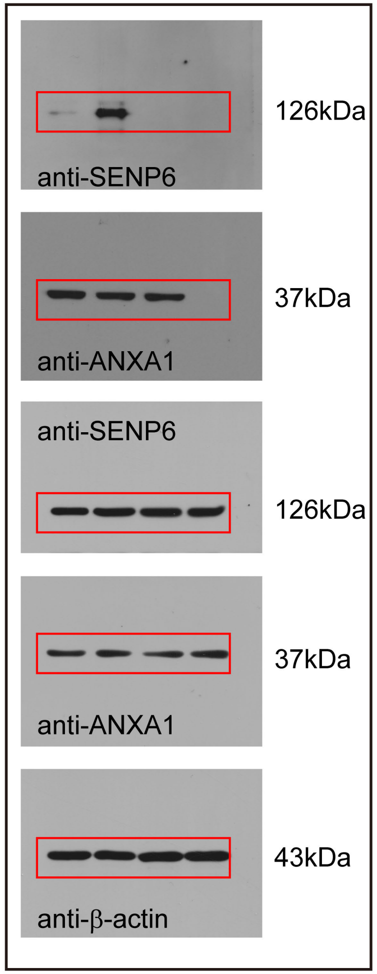

Figure 6E

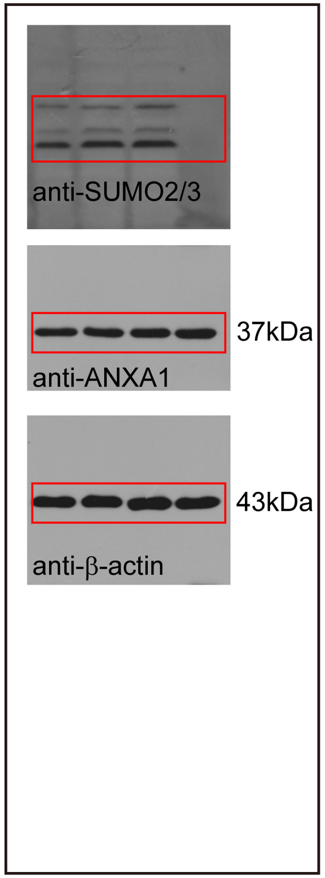

Figure 6F

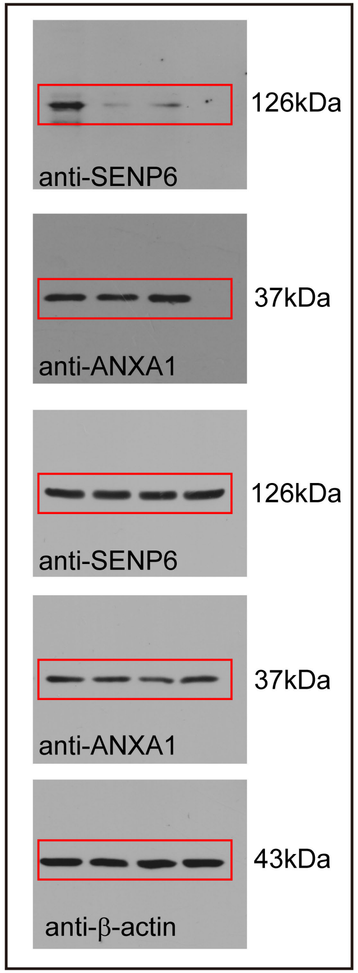

Figure 7C

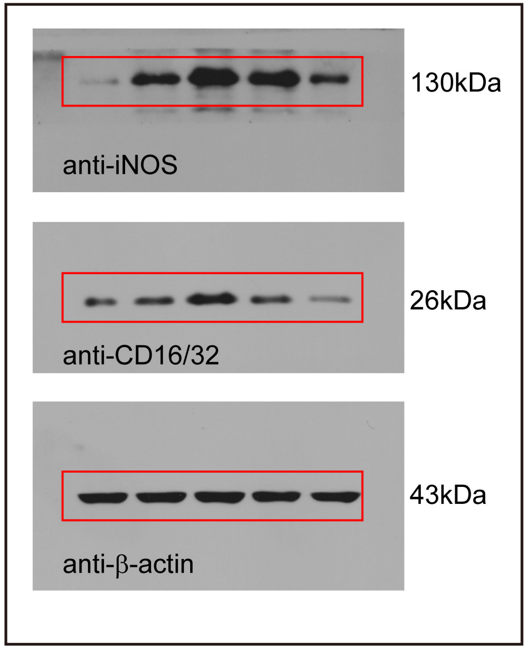

Figure 8F

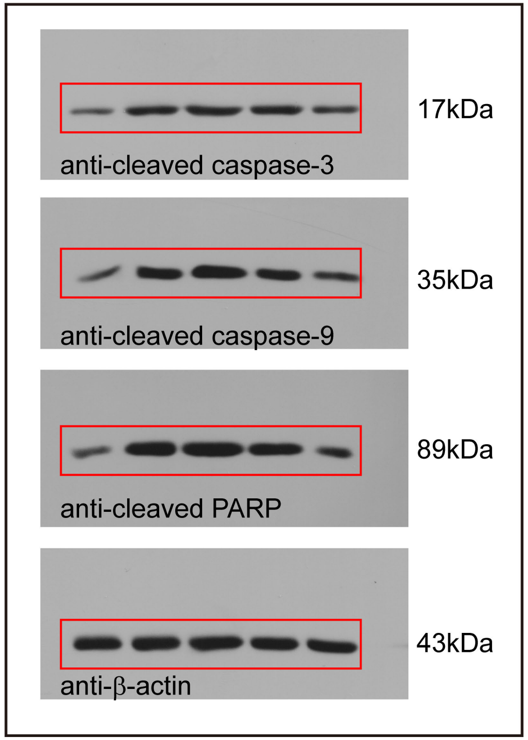

Supplementary Figure 1

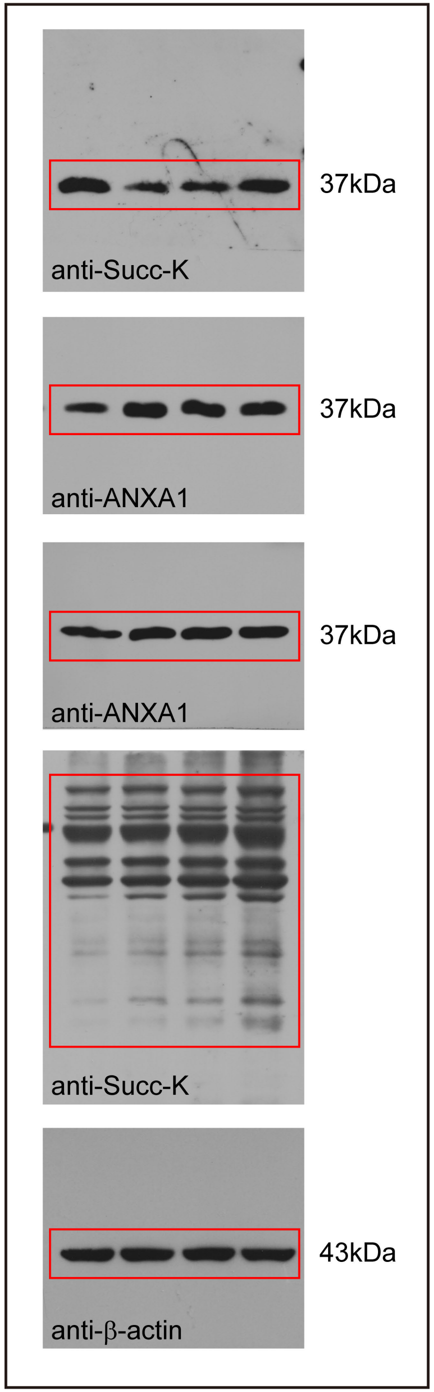

Supplement: Supplementary file 9 — Additional file 9: Full gels of the western blot images. [file 12974_2022_2665_MOESM9_ESM.pdf]
